# Unveiling the Power of Implicit Six-Point Block Scheme: Advancing numerical approximation of two-dimensional PDEs in physical systems

**DOI:** 10.1371/journal.pone.0301505

**Published:** 2024-05-16

**Authors:** Ezekiel Olaoluwa Omole, Emmanuel Olusheye Adeyefa, Kemisola Iyabo Apanpa, Victoria Iyadunni Ayodele, Femi Emmanuel Amoyedo, Homan Emadifar

**Affiliations:** 1 Department Physical Sciences, Mathematics Programme, College of Pure and Applied Sciences, Landmark University, Omu-Aran, Kwara State, Nigeria; 2 SDG 4: Quality Education Research Group, Landmark University, Omu-Aran, Nigeria; 3 Mathematics Department, Faculty of Science, Federal University Oye-Ekiti, Oye-Ekiti, Ekiti State, Nigeria; 4 Mathematics Department, Faculty of Science, University of Jos, Jos, Nigeria; 5 Computer Science & Mathematics Department, Nigeria Police Academy, Wudil-Kano, Kano State, Nigeria; 6 Department of Mathematics, Saveetha School of Engineering, Saveetha Institute of Medical and Technical Sciences, Saveetha University, Chennai, Tamil Nadu, India; 7 MEU Research Unit, Middle East University, Amman, Jordan; 8 Department of Mathematics, Hamedan Branch, Islamic Azad University, Hamedan, Iran; University of Education, PAKISTAN

## Abstract

In the era of computational advancements, harnessing computer algorithms for approximating solutions to differential equations has become indispensable for its unparalleled productivity. The numerical approximation of partial differential equation (PDE) models holds crucial significance in modelling physical systems, driving the necessity for robust methodologies. In this article, we introduce the Implicit Six-Point Block Scheme (ISBS), employing a collocation approach for second-order numerical approximations of ordinary differential equations (ODEs) derived from one or two-dimensional physical systems. The methodology involves transforming the governing PDEs into a fully-fledged system of algebraic ordinary differential equations by employing ISBS to replace spatial derivatives while utilizing a central difference scheme for temporal or *y*-derivatives. In this report, the convergence properties of ISBS, aligning with the principles of multi-step methods, are rigorously analyzed. The numerical results obtained through ISBS demonstrate excellent agreement with theoretical solutions. Additionally, we compute absolute errors across various problem instances, showcasing the robustness and efficacy of ISBS in practical applications. Furthermore, we present a comprehensive comparative analysis with existing methodologies from recent literature, highlighting the superior performance of ISBS. Our findings are substantiated through illustrative tables and figures, underscoring the transformative potential of ISBS in advancing the numerical approximation of two-dimensional PDEs in physical systems.

## 1. Background information

Partial Differential Equations (PDEs) are a useful tool for the mathematical expression of many natural phenomena and are useful in the solution of physical and other issues requiring functions of several variables. The transmission of heat/sound, fluid movement, turbulent flow, heat transfer analysis, elasticity, electrostatics, and electrodynamics are a few examples of these issues; see Ahsan *et al.* [1], Wang and Guo [2], Arif *et al.* [3, 4], Adoghe *et al.* [5], Nawaz *et al.* [6], Animasaun *et al.* [7], Devnath *et al.* [8], Ahsan *et al.* [9], Wang *et al.* [10], Rufai *et al.* [11], Nawaz and Arif [12], Ramakrishna *et al.* [13], El Misilmani *et al.* [14]). According to Quarteroni and Valli [15], numerical approximation techniques for partial differential equations (PDEs) constitute a cornerstone in diverse scientific and engineering disciplines. In agreement with Le Dret and Lucquin [16], PDEs are fundamental tools for describing the intricate behaviour of physical systems and phenomena. However, deriving analytical solutions for many PDEs poses significant challenges, often rendering such endeavours impractical or unfeasible. In this context, numerical methods emerge as indispensable tools, offering pragmatic and efficient avenues to approximate solutions of a well-known second-order PDEs arising from physical system with a rectangular domain Γ = {(*x*, *y*):(*x*, *y*) ∈ [*a*, *b*] × [*c*, *d*]} given by Eq (1) below. Eq (1) embodies a versatile framework applicable across diverse fields within contemporary society. Its utility extends to the design and optimization of a wide array of systems, spanning from aerospace engineering (including aircraft and submarines) to biological and chemical processes. Additionally, these equations find application in mathematical models encompassing viscoelastic flows, various dynamic systems, medical imaging technologies, and the development of pharmaceuticals; see Bergounioux *et al.* [17]. The study of numerical solutions for Partial Differential Equations (PDEs) has yielded crucial insights across diverse fields such as photo-acoustic tomography, gas dynamics, aerodynamic shape optimization, and flow control design. Gunzburger [18] and Bredies *et al.* [19] underscored the significance of approximate PDE solutions. In the realms of physical sciences, applied mathematics, engineering, and economics, inherent physical constraints often necessitate the utilization of PDEs for modelling. Neittaanmaki and Tiba [20] discuss controlling difficulties, mainly when specific parameters, conditions, or constraints govern these PDEs throughout the computation. Given the nonlinear nature of the majority of these governing equations, as highlighted by Leugering *et al.* [21] and Aubert and Kornprobst [22], obtaining analytical solutions via theoretical methods proves exceedingly challenging.

$$\begin{array}{c} a(x,y)\frac{\partial^{2}u}{\partial x^{2}}+b(x,y)\frac{\partial^{2}u}{\partial y^{2}}+p(x,y)\frac{\partial u}{\partial x}+r(x,y)\frac{\partial u}{\partial y}+k(x,y)u=g(x,y). \end{array}$$ (1)

Building upon the insights of Debnath [23], the practicality of using computers to generate approximate solutions becomes evident, rendering it desirable and imperative to explore control and optimization techniques for various PDE models. Among them is time-dependent (PDE)-driven optimal control problems which have recently garnered significant attention within the scientific computing community due to their numerical complexities. Numerous methods have emerged in literature aimed at approximating the solutions to problems described by Eq (1) under various conditions. For instance, Liu *et al.* [24] introduced a novel hybrid approach combining Haar wavelets and finite differences to tackle the hyperbolic Schrodinger Equation incorporating a nonlinear function, energy, and mass conversion terms. Their study included a rigorous convergence analysis of the hybrid method, supported by illustrative curves facilitating clear comprehension and interpretation of the results. In another study, Raslan *et al.* [25] devised an extended version of cubic B-splines in n-dimensional space specifically tailored for the numerical treatment of PDEs, with notable applications. The results of the study demonstrated a commendable level of efficiency and accuracy, particularly evident in terms of convergence characteristics. Mirzaee *et al.* [26] showcased the versatility of meshfree and finite difference methods in tackling the stochastic time-fractional sine-Gordon equation within two-dimensional space, particularly on non-rectangular domains. Meanwhile, Lakestani and Dehghan [27] presented a meticulous numerical solution to the Nonlinear Klein-Gordon Equation, employing a blend of collocation and finite difference-collocation techniques. In a distinct avenue, Shiralashetti *et al.* [28] delved into the realm of PDE application problems, specifically focusing on elasto-hydrodynamic lubrication issues, and explored the efficacy of biorthogonal wavelet-based full-approximation techniques. Moreover, the discussion by Juraev and Gasimov [29] delved into the intricacies of the Cauchy issue associated with matrix factorizations of the Helmholtz equation, shedding light on regularization techniques within multidimensional bounded domains.

Predictor-corrector methods are numerical techniques used for solving boundary value problems (BVPs) of ordinary differential equations (ODEs). As presented by Butcher and Wanner [30] an initial approximation to the solution is made using a simple numerical method like Euler’s method or the Runge-Kutta method and such approximation is referred to as the “predictor.” Sequel to Gragg, W. B., & Stetter [31], the predictor step provides an initial estimate of the solution. In the correction step, this estimate is refined using a more accurate numerical method, typically a higher-order method like the Adams-Bashforth or Adams-Moulton methods and such a refined estimate is called the “corrector.” Based on Garrido *et al.* [32], worth remarking that the predictor and corrector steps are often applied iteratively until the solution converges to the desired accuracy or until a specified stopping criterion is met. Following Diamantakis *et al.* [33], since predictor-corrector methods are used for boundary value problems, the boundary conditions are incorporated into the iterative process to ensure that the solution satisfies the given boundary conditions. Worth concluding that the convergence and stability of predictor-corrector methods depend on factors such as the choice of predictor and corrector methods, step size, and properties of the differential equation being solved. Similarly, many authors have explored employing predictor-corrector methodologies for the numerical resolution of boundary value problems or differential equations encompassing initial conditions. For instance, Su and Zhou [34], Shokri and Saadat [35], and Awoyemi [36] have contributed to this discourse. Sunday *et al.* [37] subsequently pioneered a suite of off-step models facilitated by a self-starting technique, leveraging these models to tackle many consequential application equations, such as the Kepler Problem. Ramos and Vigo-Aguiar [38] have also conducted seminal research on BDF-style equations tailored for integrating a class of stiff problems, employing the L-stable technique of lines. Ngwane and Jator [39] employed the sophisticated trigonometrically fitted block technique to tackle a complex oscillating system of equations, exhibiting both Hamiltonian dynamics and second-order initial conditions. Modebei *et al.* [40] proposed a novel numerical approach for simulating fourth-order differential equations involving partial derivatives, leveraging uniform-order block formulae outlined in their reference. Jator [41] delved into research on block algorithms to provide precise and efficient solutions for sine-Gordon partial differential equations (PDEs) with varying parameters. Olaiya et al. [42] scrutinized the numerical models utilized in resolving the solutions for the Black-Scholes differential equation. Additionally, Familua *et al.* [43] conducted an in-depth examination of advanced self-starting algorithms tailored for numerically simulating differential equations featuring second derivatives, offering diverse practical applications.

As demonstrated by Farkas and Deconinck [44], Animasaun et al. [45], the numerical solution of the Heat Equation provide invaluable insights into the behaviour of heat transfer phenomena in various physical systems, guiding the design of efficient thermal management solutions crucial for industries ranging from electronics to aerospace engineering. Worth tracing to Ullah *et al.* [46] and Salahudin *et al.* [47] that understanding the numerical solution of the Wave Equation illuminates the propagation of waves in diverse mediums, offering indispensable tools for predicting seismic activity, designing telecommunications networks, and optimizing acoustic environments in architectural and industrial settings. As presented by Rehman *et al.* [48] and Khan *et al.* [49], the numerical solution of the Schrodinger Equation underpins quantum mechanical simulations, empowering scientists to unravel the intricate behaviour of fundamental particles and molecules, with applications spanning from drug discovery to the development of quantum computing algorithms. The computational exploration of the Navier-Stokes Equations was shown by Johnson [50], Fisher *et al.* [51], and Jameson *et al.* [52] that its solution is capable of unlocking the complexities of fluid dynamics, enabling advancements in areas crucial to human civilization, such as weather forecasting, aerodynamics, and the optimization of industrial processes, from energy production to transportation systems. This report unveils the findings of an intensive research endeavour aimed at unleashing the potential of implicit six-point block schemes in revolutionizing the numerical approximation of two-dimensional Partial Differential Equations (PDEs) within physical systems. These block solvers not only retain the inherent self-starting nature of algorithms but also exhibit rapid convergence rates and a unique ability to provide accurate approximations across various stages of computation. Non-linear partial differential equations are omnipresent in science and engineering, with notable examples including the Helmholtz and convection-diffusion equations. Despite the pivotal role implicit block methods play in tackling these equations, a conspicuous need for more attention is directed towards developing efficient numerical techniques grounded in these methods for solving (1). A profound understanding of their intricate dynamics is imperative to engineer mathematical methodologies that seamlessly amalgamate precision with efficiency. Hence, we propose the introduction of a seventh-order Implicit Six-point Block Scheme (ISBS). Employing the ISBS holds the promise of delivering more accurate solutions with accelerated convergence rates for these equations. Remarkably, the methodology being developed in this study boasts significant computational prowess and exhibits a wide array of applications, surpassing existing methods delineated in the literature.

### 2. Development of the Implicit Six-Point Block Scheme (ISBS)

Hermite polynomials are orthogonal with respect to the weight function $e^{-x^{2}}$ on the interval (−∞, ∞). According to Thakare *et al.* [53], Adeyefa *et al.* [54], and recently by Dattoli and Licciardi [55], it is worth remarking that this orthogonality property simplifies many computations, especially when dealing with integrals or solving differential equations. Hermite polynomials, which belong to the class of orthogonal polynomials, are defined using recurrence formulas on the interval (−∞, ∞) as

$$\begin{array}{c} \tau_{n+1}(x)=x\tau_{n}\left(x\right)-\tau_{n}^{\prime }\left(x\right) \end{array}$$ (2)

In relation to the weight function $e^{-x^{2}}$, the polynomials are orthonormal. According to Salzer *et al.* [56], the first four Orthogonal Hermite polynomias are,

$$\begin{array}{c} \tau_{0}\left(x\right)=1,\;\;\;\tau_{1}\left(x\right)=x,\;\;\;\tau_{2}\left(x\right)=x^{2}-1,\;\;\;\tau_{3}\left(x\right)=x^{3}-3x \end{array}$$ (3)

### 2.1 Development of the method

Consider the partial sum of the Hermite approximation defined as

$$\begin{array}{c} U(x)=\sum_{r=0}^{m+n-1}\zeta_{r}\tau_{r}(x),\; \end{array}$$ (4)

Differentiating Eq (4) twice to obtain

$$\begin{array}{c} U^{\prime \prime }(x)=\sum_{r=0}^{m+n-1}\zeta_{r}\tau_{r}^{\prime \prime }(x),\;r\in \left(0,6\right)\; \end{array}$$ (5)

Next is to input *m* = 6, *n* = 2, and *k*, where *k* is the step number. As a result, m is the number of selected collocation points, and n is the number of individual interpolation points required to suit the order of the partial differential equation in (1). Eqs (4) and (5) thus decrease to,

$$\begin{array}{c} U(x)=\sum_{r=0}^{8}\zeta_{r}\tau_{r}(x),\; \end{array}$$ (6)

Similarly, differentiating (6) twice gives;

$$\begin{array}{c} U^{\prime \prime }(x)=\sum_{r=0}^{8}\zeta_{r}\tau_{r}^{\prime \prime }(x),\; \end{array}$$ (7)

Right now After collocating the differential system (7) at *x*_*n*+*w*_, *w* = 0(1)6 and interpolating the approximate solution (6) at *x*_*n*+*w*_, *w* = 0(1), seven equations are produced. The following is an expression for these equations as a matrix system of equations,

$$\begin{array}{c} \Theta J=Z; \end{array}$$ (8)

assume,

$$\begin{array}{c} \Theta =[\begin{array}{ccccccc} \tau_{0}\left(x_{n}\right) & \tau_{1}\left(x_{n}\right) & \tau_{2}\left(x_{n}\right) & \tau_{3}\left(x_{n}\right) & \cdots & \tau_{k+2}\left(x_{n}\right) & \\ \tau_{0}\left(x_{n+1}\right) & \tau_{1}\left(x_{n+1}\right) & \tau_{2}\left(x_{n+1}\right) & \tau_{0}\left(x_{n+1}\right) & \cdots & \tau_{k+2}\left(x_{n+1}\right) & \\ \tau_{0}^{\prime \prime }\left(x_{n}\right) & \tau_{1}^{\prime \prime }\left(x_{n}\right) & \tau_{2}^{\prime \prime }\left(x_{n}\right) & \tau_{3}^{\prime \prime }\left(x_{n}\right) & \cdots & \tau_{k+2}^{\prime \prime }\left(x_{n}\right) & \\ \tau_{0}^{\prime \prime }\left(x_{n+1}\right) & \tau_{1}^{\prime \prime }\left(x_{n+1}\right) & \tau_{2}^{\prime \prime }\left(x_{n+1}\right) & \tau_{3}^{\prime \prime }\left(x_{n+1}\right) & \cdots & \tau_{k+2}^{\prime \prime }\left(x_{n+1}\right) & \\ \vdots & \vdots & \vdots & \vdots & \cdots & \vdots \\ \vdots & \vdots & \vdots & \vdots & \cdots & \vdots \\ \tau_{0}^{\prime \prime }\left(x_{n+k}\right) & \tau_{1}^{\prime \prime }\left(x_{n+k}\right) & \tau_{2}^{\prime \prime }\left(x_{n+k}\right) & \tau_{3}^{\prime \prime }\left(x_{n+k}\right) & \cdots & \tau_{k+2}^{\prime \prime }\left(x_{n+k}\right) \end{array}]\; \end{array}$$

$$\begin{array}{c} J={\left[\begin{array}{c} \zeta_{0},\zeta_{1},\zeta_{2},\zeta_{3},\cdots ,\zeta_{8} \end{array}\right]}^{T},\;\;Z={\left[\begin{array}{c} u_{m,n},u_{m+1,n},\eta_{m,n},\eta_{m+1,n},\cdots ,\eta_{m+6,n} \end{array}\right]}^{T} \end{array}$$

By applying the matrix inverse approach to solve the matrix Eq (8) for the unknown coefficients of *ζ*_*i*_, *i* = 0(1)8, where *J* = Θ^−1^*Z*, or with the use of computer-aided tools like Mathematica 11.0. The obtained values are then used to replace (6) and set *x* = *ϕh*+*x*_*n*+5_ in order to get the form’s continuous function;

$$\begin{array}{ccc} u_{m+j,n}(\phi ) & = & \Psi_{0}u_{m,n}+\Psi_{1}u_{m+1,n}+h^{2}\sum_{j=0}^{6}\Delta_{j}(\phi )\eta_{m+j,n},\;\;j=0(1)6 \end{array}$$ (9)

the matrix-formatted coefficients of the continuous function (9) are shown below.

$$\begin{array}{c} \left[\begin{array}{c} \Psi_{0}\\ \Psi_{1} \end{array}]=[\begin{array}{cc} -1 & -4\\ 1 & 5 \end{array}][\begin{array}{c} \phi^{0}\\ \phi^{1} \end{array}\right] \end{array}$$ (10)

$$\begin{array}{c} \left[\begin{array}{c} \Delta_{0}\\ \Delta_{1}\\ \Delta_{2}\\ \Delta_{3}\\ \Delta_{4}\\ \Delta_{5}\\ \Delta_{6} \end{array}]=[\begin{array}{cccccccc} \frac{1669}{6048} & \frac{2867}{40320} & -\frac{1}{180} & -\frac{13}{4320} & \frac{1}{960} & \frac{1}{864} & \frac{1}{3360} & \frac{1}{40320}\\ \frac{3875}{1008} & \frac{3875}{4032} & \frac{1}{24} & \frac{31}{1440} & -\frac{1}{120} & -\frac{1}{120} & -\frac{1}{504} & -\frac{1}{6720}\\ \frac{5069}{2016} & \frac{38401}{40320} & -\frac{5}{36} & -\frac{19}{288} & \frac{29}{960} & \frac{37}{1440} & \frac{11}{2016} & \frac{1}{2688}\\ \frac{3751}{1512} & \frac{1399}{1440} & \frac{5}{18} & \frac{47}{432} & \frac{-1}{15} & -\frac{23}{540} & -\frac{1}{126} & -\frac{1}{2016}\\ \frac{1457}{2016} & \frac{46453}{40320} & -\frac{5}{12} & -\frac{17}{288} & \frac{83}{960} & \frac{19}{480} & \frac{13}{2016} & \frac{1}{2688}\\ \frac{179}{1008} & \frac{8191}{20160} & \frac{77}{360} & -\frac{49}{1440} & -\frac{7}{120} & -\frac{7}{360} & -\frac{1}{360} & -\frac{1}{6720}\\ -\frac{95}{6048} & -\frac{13}{896} & \frac{1}{36} & \frac{137}{4320} & \frac{1}{64} & \frac{17}{4320} & \frac{1}{2016} & \frac{1}{40320} \end{array}][\begin{array}{c} \phi^{0}\\ \phi^{1}\\ \phi^{3}\\ \phi^{4}\\ \phi^{5}\\ \phi^{6}\\ \phi^{7}\\ \phi^{8} \end{array}\right] \end{array}$$ (11)

Evaluating (9) at *ϕ* = −3, −2, −1, 0, and 1 yields the following discrete schemes, which are constructed as the main discrete scheme.

$$\begin{array}{ccc} u_{m+2,n} & = & 2\;u_{m+1,n}-u_{m,n}+\frac{863}{12096}\;h^{2}\eta_{m,n}+\frac{8999}{10080}\;h^{2}\eta_{m+1,n}-\frac{769}{20160}\;h^{2}\eta_{m+2,n}+\frac{1987}{15120}\;h^{2}\eta_{m+3,n}\\ & & -\frac{1609}{20160}\;h^{2}\eta_{m+4,n}+\frac{263}{10080}\;h^{2}\eta_{m+5,n}-\frac{221}{60480}\;h^{2}\eta_{m+6,n} \end{array}$$ (12)

$$\begin{array}{ccc} u_{m+3,n} & = & 3\;u_{m+1,n}-2\;u_{m,n}+\frac{2803}{20160}\;h^{2}\eta_{m,n}+\frac{1265}{672}\;h^{2}\eta_{m+1,n}+\frac{1657}{2240}\;h^{2}\eta_{m+2,n}+\frac{1777}{5040}\;h^{2}\eta_{m+3,n}\\ & & -\frac{1049}{6720}\;h^{2}\eta_{m+4,n}+\frac{11}{224}\;h^{2}\eta_{m+5,n}-\frac{137}{20160}\;h^{2}\eta_{m+6,n} \end{array}$$ (13)

$$\begin{array}{ccc} u_{m+4,n} & = & 4\;u_{m+1,n}-3\;u_{m,n}+\frac{2089}{10080}\;h^{2}\eta_{m,n}+\frac{4813}{1680}\;h^{2}\eta_{m+1,n}+\frac{5461}{3360}\;h^{2}\eta_{m+2,n}+\frac{3457}{2520}\;h^{2}\eta_{m+3,n}\\ & & -\frac{419}{3360}\;h^{2}\eta_{m+4,n}+\frac{109}{1680}\;h^{2}\eta_{m+5,n}-\frac{19}{2016}\;h^{2}\eta_{m+6,n} \end{array}$$ (14)

$$\begin{array}{ccc} u_{m+5,n} & = & +5\;u_{m+1,n}-4\;u_{m,n}+\frac{1669}{6048}\;h^{2}\eta_{m,n}+\frac{3875}{1008}\;h^{2}\eta_{m+1,n}+\frac{5069}{2016}\;h^{2}\eta_{m+2,n}+\frac{3751}{1512}\;h^{2}\eta_{m+3,n}\\ & & +\frac{1457}{2016}\;h^{2}\eta_{m+4,n}+\frac{179}{1008}\;h^{2}\eta_{m+5,n}-\frac{95}{6048}\;h^{2}\eta_{m+6,n} \end{array}$$ (15)

$$\begin{array}{ccc} u_{m+6,n} & = & 6\;u_{m+1,n}-5\;u_{m,n}+\frac{1375}{4032}\;h^{2}\eta_{m,n}+\frac{3259}{672}\;h^{2}\eta_{m+1,n}+\frac{1489}{448}\;h^{2}\eta_{m+2,n}+\frac{3751}{1008}\;h^{2}\eta_{m+3,n}\\ & & +\frac{2059}{1344}\;h^{2}\eta_{m+4,n}+\frac{265}{224}\;h^{2}\eta_{m+5,n}+\frac{199}{4032}\;h^{2}\eta_{m+6,n} \end{array}$$ (16)

Below is the first derivative of (9),

$$\begin{array}{ccc} u_{m+j,n}^{\prime }(t) & = & \Psi_{0}^{\prime }u_{m,n}+\Psi_{1}^{\prime }u_{m+1,n}+h^{2}\sum_{j=0}^{6}\Delta_{j}^{\prime }(\phi )\eta_{m+j,n},\;\;j=0(1)6 \end{array}$$ (17)

The function (17) has coefficients that are the first derivative of (10) and (11), which are as follows,

$$\begin{array}{c} \left[\begin{array}{c} \Psi_{0}^{\prime }\\ \Psi_{1}^{\prime } \end{array}]=[\begin{array}{c} -1\\ 1 \end{array}][\begin{array}{c} \phi^{1} \end{array}\right] \end{array}$$ (18)

$$\begin{array}{c} \left[\begin{array}{c} \Delta_{0}^{\prime }\\ \Delta_{1}^{\prime }\\ \Delta_{2}^{\prime }\\ \Delta_{3}^{\prime }\\ \Delta_{4}^{\prime }\\ \Delta_{5}^{\prime }\\ \Delta_{6}^{\prime } \end{array}]=[\begin{array}{ccccccc} \frac{2867}{40320} & -\frac{1}{60} & -\frac{13}{1080} & \frac{1}{192} & \frac{1}{144} & \frac{1}{480} & \frac{1}{5040}\\ \frac{3875}{4032} & \frac{1}{8} & \frac{31}{360} & -\frac{1}{24} & -\frac{1}{20} & -\frac{1}{72} & -\frac{1}{840}\\ \frac{38401}{40320} & -\frac{5}{12} & -\frac{19}{72} & \frac{29}{192} & \frac{37}{240} & \frac{11}{288} & \frac{1}{336}\\ \frac{1399}{1440} & \frac{5}{6} & \frac{47}{108} & -\frac{1}{3} & -\frac{23}{90} & -\frac{1}{18} & -\frac{1}{252}\\ \frac{46453}{40320} & -\frac{5}{4} & -\frac{17}{72} & \frac{83}{192} & \frac{19}{80} & \frac{13}{288} & \frac{1}{336}\\ \frac{8191}{20160} & \frac{77}{120} & -\frac{49}{360} & -\frac{7}{24} & -\frac{7}{60} & -\frac{7}{360} & -\frac{1}{840}\\ -\frac{13}{896} & \frac{1}{12} & \frac{137}{1080} & \frac{5}{64} & \frac{17}{720} & \frac{1}{288} & \frac{1}{5040} \end{array}][\begin{array}{c} \phi^{0}\\ \phi^{2}\\ \phi^{3}\\ \phi^{4}\\ \phi^{5}\\ \phi^{6}\\ \phi^{7} \end{array}\right] \end{array}$$ (19)

By evaluating (17) at the locations *ϕ* = −5, −4, −3, −2, −1, 0, and 1, the additional discrete scheme is produced. The first derivative discrete schemes that result are as follows,

$$\begin{array}{ccc} {u^{\prime }}_{m,n} & = & -\frac{1}{120960h}\;(28549\;h^{2}\eta_{m,n}+57750\;h^{2}\eta_{m+1,n}-51453\;h^{2}\eta_{m+2,n}+42484\;h^{2}\eta_{m+3,n}\\ & & -23109\;h^{2}\eta_{m+4,n}+7254\;h^{2}\eta_{m+5,n}-995\;h^{2}\eta_{m+6,n}+120960\;u_{m,n}-120960\;u_{m+1,n}) \end{array}$$ (20)

$$\begin{array}{ccc} {u^{\prime }}_{m+1,n} & = & \frac{1}{120960h}\;(9625\;h^{2}\eta_{m,n}+72474\;h^{2}\eta_{m+1,n}-41469\;h^{2}\eta_{m+2,n}+32524\;h^{2}\eta_{m+3,n}\\ & & -17313\;h^{2}\eta_{m+4,n}+5370\;h^{2}\eta_{m+5,n}-731\;h^{2}\eta_{m+6,n}-120960\;u_{m,n}+120960\;u_{m+1,n}) \end{array}$$ (21)

$$\begin{array}{ccc} {u^{\prime }}_{m+2,n} & = & \frac{1}{40320h}\;(2633\;h^{2}\eta_{m,n}+40910\;h^{2}\eta_{m+1,n}+17503\;h^{2}\eta_{m+2,n}+4\;h^{2}\eta_{m+3,n}-905\;h^{2}\eta_{m+4,n}\\ & & +398\;h^{2}\eta_{m+5,n}-63\;h^{2}\eta_{m+6,n}-40320\;u_{m,n}+40320\;u_{m+1,n}) \end{array}$$ (22)

$$\begin{array}{ccc} {u^{\prime }}_{m+3,n} & = & \frac{1}{120960h}\;(8441\;h^{2}\eta_{m,n}+117210\;h^{2}\eta_{m+1,n}+114147\;h^{2}\eta_{m+2,n}+75020\;h^{2}\eta_{m+3,n}\\ & & -16257\;h^{2}\eta_{m+4,n}+4410\;h^{2}\eta_{m+5,n}-571\;h^{2}\eta_{m+6,n}-120960\;u_{m,n}+120960\;u_{m+1,n}) \end{array}$$ (23)

$$\begin{array}{ccc} {u^{\prime }}_{m+4,n} & = & \frac{1}{120960h}\;(8059\;h^{2}\eta_{m,n}+120426\;h^{2}\eta_{m+1,n}+100605\;h^{2}\eta_{m+2,n}+150028\;h^{2}\eta_{m+3,n}\\ & & +45381\;h^{2}\eta_{m+4,n}-1110\;h^{2}\eta_{m+5,n}-29\;h^{2}\eta_{m+6,n}-120960\;u_{m,n}+120960\;u_{m+1,n}) \end{array}$$ (24)

$$\begin{array}{ccc} {u^{\prime }}_{m+5,n} & = & \frac{1}{40320h}\;(2867\;h^{2}\eta_{m,n}+38750\;h^{2}\eta_{m+1,n}+38401\;h^{2}\eta_{m+2,n}+39172\;h^{2}\eta_{m+3,n}\\ & & +46453\;h^{2}\eta_{m+4,n}+16382\;h^{2}\eta_{m+5,n}-585\;h^{2}\eta_{m+6,n}-40320\;u_{m,n}+40320\;u_{m+1,n}) \end{array}$$ (25)

$$\begin{array}{ccc} {u^{\prime }}_{m+6,n} & = & \frac{1}{120960h}\;(6875\;h^{2}\eta_{m,n}+128874\;h^{2}\eta_{m+1,n}+74781\;h^{2}\eta_{m+2,n}+192524\;h^{2}\eta_{m+3,n}\\ & & +46437\;h^{2}\eta_{m+4,n}+179370\;h^{2}\eta_{m+5,n}+36419\;h^{2}\eta_{m+6,n}-120960\;u_{m,n}+120960\;u_{m+1,n}) \end{array}$$ (26)

### 2.2 Formulation of Implicit Six-point Block Scheme (ISBS)

By joining the discrete schemes (12)–(16) and derivative (20) at *x*_*m*,*n*_ formed matrix equation below,

$$\begin{array}{c} EU_{m}=F\lambda_{0}+G\lambda_{1}+h^{2}\left[H\lambda_{2}+I\lambda_{3}\right] \end{array}$$ (27)

$$\begin{array}{c} E=[\begin{array}{cccccc} -120960 & 60480 & 0 & 0 & 0 & 0\\ -60480 & 0 & 20160 & 0 & 0 & 0\\ -40320 & 0 & 0 & 10080 & 0 & 0\\ -30240 & 0 & 0 & 0 & 6048 & 0\\ -24192 & 0 & 0 & 0 & 0 & 4032\\ -120960 & 0 & 0 & 0 & 0 & 0 \end{array}],U_{m}=[\begin{array}{c} u_{m+1,n}\\ u_{m+2,n}\\ u_{m+3,n}\\ u_{m+4,n}\\ u_{m+5,n}\\ u_{m+6,n} \end{array}] \end{array}$$

$$\begin{array}{c} F=[\begin{array}{cccccc} 0 & 0 & 0 & 0 & 0 & -60480\\ 0 & 0 & 0 & 0 & 0 & -40320\\ 0 & 0 & 0 & 0 & 0 & -30240\\ 0 & 0 & 0 & 0 & 0 & -24192\\ 0 & 0 & 0 & 0 & 0 & -20160\\ 0 & 0 & 0 & 0 & 0 & -120960 \end{array}],\lambda_{0}=[\begin{array}{c} u_{m-1,n}\\ u_{m-2,n}\\ u_{m-3,n}\\ u_{m-4,n}\\ u_{m-5,n}\\ u_{m,n} \end{array}] \end{array}$$

$$\begin{array}{c} G=[\begin{array}{cccccc} 0 & 0 & 0 & 0 & 0 & 0\\ 0 & 0 & 0 & 0 & 0 & 0\\ 0 & 0 & 0 & 0 & 0 & 0\\ 0 & 0 & 0 & 0 & 0 & 0\\ 0 & 0 & 0 & 0 & 0 & 0\\ 0 & 0 & 0 & 0 & 0 & -120960 \end{array}],\lambda_{1}=[\begin{array}{c} u_{m-1,n}^{\prime }\\ u_{m-2,n}^{\prime }\\ u_{m-3,n}^{\prime }\\ u_{m-4,n}^{\prime }\\ u_{m-5,n}^{\prime }\\ u_{m,n}^{\prime } \end{array}] \end{array}$$

$$\begin{array}{c} H=[\begin{array}{cccccc} 0 & 0 & 0 & 0 & 0 & 4315\\ 0 & 0 & 0 & 0 & 0 & 2803\\ 0 & 0 & 0 & 0 & 0 & 2089\\ 0 & 0 & 0 & 0 & 0 & 1669\\ 0 & 0 & 0 & 0 & 0 & 1375\\ 0 & 0 & 0 & 0 & 0 & -28549 \end{array}],\lambda_{2}=[\begin{array}{c} \eta_{m-1,n}\\ \eta_{m-2,n}\\ \eta_{m-3,n}\\ \eta_{m-4,n}\\ \eta_{m-5,n}\\ \eta_{m,n} \end{array}] \end{array}$$

$$\begin{array}{c} I=[\begin{array}{cccccc} 53994 & -2307 & 7948 & -4827 & 1578 & -221\\ 37950 & 14913 & 7108 & -3147 & 990 & -137\\ 28878 & 16383 & 13828 & -1257 & 654 & -95\\ 23250 & 15207 & 15004 & 4371 & 1074 & -95\\ 19554 & 13401 & 15004 & 6177 & 4770 & 199\\ -57750 & 51453 & -42484 & 23109 & -7254 & 995 \end{array}],\lambda_{3}=[\begin{array}{c} \eta_{m+1,n}\\ \eta_{m+2,n}\\ \eta_{m+3,n}\\ \eta_{m+4,n}\\ \eta_{m+5,n}\\ \eta_{m+6,n} \end{array}] \end{array}$$

The product of the matrix Eq (27) with the inverse *E* gives the Implicit Six-point Block Scheme of the form

$$\begin{array}{c} U_{m}=\bar{F}\lambda_{0}+\bar{G}\lambda_{1}+h^{2}\left[\bar{H}\lambda_{2}+\bar{I}\lambda_{3}\right] \end{array}$$ (28)

$$\begin{array}{c} U_{m}=[\begin{array}{cccccc} 1 & 0 & 0 & 0 & 0 & 0\\ 0 & 1 & 0 & 0 & 0 & 0\\ 0 & 0 & 1 & 0 & 0 & 0\\ 0 & 0 & 0 & 1 & 0 & 0\\ 0 & 0 & 0 & 0 & 1 & 0\\ 0 & 0 & 0 & 0 & 0 & 1 \end{array}],\bar{F}=[\begin{array}{cccccc} 0 & 0 & 0 & 0 & 0 & 1\\ 0 & 0 & 0 & 0 & 0 & 1\\ 0 & 0 & 0 & 0 & 0 & 1\\ 0 & 0 & 0 & 0 & 0 & 1\\ 0 & 0 & 0 & 0 & 0 & 1\\ 0 & 0 & 0 & 0 & 0 & 1 \end{array}] \end{array}$$

$$\begin{array}{c} \bar{G}=[\begin{array}{cccccc} 0 & 0 & 0 & 0 & 0 & 1\\ 0 & 0 & 0 & 0 & 0 & 2\\ 0 & 0 & 0 & 0 & 0 & 3\\ 0 & 0 & 0 & 0 & 0 & 4\\ 0 & 0 & 0 & 0 & 0 & 5\\ 0 & 0 & 0 & 0 & 0 & 6 \end{array}]\bar{H}=[\begin{array}{cccccc} 0 & 0 & 0 & 0 & 0 & \frac{28549}{120960}\\ 0 & 0 & 0 & 0 & 0 & \frac{1027}{1890}\\ 0 & 0 & 0 & 0 & 0 & \frac{759}{896}\\ 0 & 0 & 0 & 0 & 0 & \frac{1088}{945}\\ 0 & 0 & 0 & 0 & 0 & \frac{35225}{24192}\\ 0 & 0 & 0 & 0 & 0 & \frac{123}{70} \end{array}] \end{array}$$

$$\begin{array}{c} \bar{I}=[\begin{array}{cccccc} \frac{275}{576} & -\frac{5717}{13440} & \frac{10621}{30240} & -\frac{7703}{40320} & \frac{403}{6720} & -\frac{199}{24192}\\ \frac{194}{105} & -\frac{8}{9} & \frac{788}{945} & -\frac{97}{210} & \frac{46}{315} & -\frac{19}{945}\\ \frac{1485}{448} & -\frac{2403}{4480} & \frac{45}{32} & -\frac{3267}{4480} & \frac{513}{2240} & -\frac{141}{4480}\\ \frac{1504}{315} & -\frac{8}{105} & \frac{2624}{945} & -\frac{8}{9} & \frac{32}{105} & -\frac{8}{189}\\ \frac{8375}{1344} & \frac{3125}{8064} & \frac{25625}{6048} & -\frac{625}{2688} & \frac{275}{576} & -\frac{1375}{24192}\\ \frac{54}{7} & \frac{27}{35} & \frac{204}{35} & \frac{27}{70} & \frac{54}{35} & 0 \end{array}] \end{array}$$

Writing out the matrix Eq (28) in a simple form as follows

$$\begin{array}{ccc} u_{m+1,n} & = & u_{m,n}+{u^{\prime }}_{m,n}h+\frac{28549}{120960}\;h^{2}\eta_{m,n}+\frac{275}{576}\;h^{2}\eta_{m+1,n}-\frac{5717}{13440}\;h^{2}\eta_{m+2,n}+\frac{10621}{30240}\;h^{2}\eta_{m+3,n}\\ & & -\frac{7703}{40320}\;h^{2}\eta_{m+4,n}+\frac{403}{6720}\;h^{2}\eta_{m+5,n}-\frac{199}{24192}\;h^{2}\eta_{m+6,n} \end{array}$$ (29)

$$\begin{array}{ccc} u_{m+2,n} & = & u_{m,n}+2h{u^{\prime }}_{m,n}+\frac{1027}{1890}\;h^{2}\eta_{m,n}+\frac{194}{105}\;h^{2}\eta_{m+1,n}-\frac{8}{9}\;h^{2}\eta_{m+2,n}+\frac{788}{945}\;h^{2}\eta_{m+3,n}\\ & & -\frac{97}{210}\;h^{2}\eta_{m+4,n}+\frac{46}{315}\;h^{2}\eta_{m+5,n}-\frac{19}{945}\;h^{2}\eta_{m+6,n} \end{array}$$ (30)

$$\begin{array}{ccc} u_{m+3,n} & = & u_{m,n}+3h{u^{\prime }}_{m,n}+\frac{759}{896}\;h^{2}\eta_{m,n}+\frac{1485}{448}\;h^{2}\eta_{m+1,n}-\frac{2403}{4480}\;h^{2}\eta_{m+2,n}+\frac{45}{32}\;h^{2}\eta_{m+3,n}\\ & & -\frac{3267}{4480}\;h^{2}\eta_{m+4,n}+\frac{513}{2240}\;h^{2}\eta_{m+5,n}-\frac{141}{4480}\;h^{2}\eta_{m+6,n} \end{array}$$ (31)

$$\begin{array}{ccc} u_{m+4,n} & = & u_{m,n}+4h{u^{\prime }}_{m,n}+\frac{1088}{945}\;h^{2}\eta_{m,n}+\frac{1504}{315}\;h^{2}\eta_{m+1,n}-\frac{8}{105}\;h^{2}\eta_{m+2,n}+\frac{2624}{945}\;h^{2}\eta_{m+3,n}\\ & & -\frac{8}{9}\;h^{2}\eta_{m+4,n}+\frac{32}{105}\;h^{2}\eta_{m+5,n}-\frac{8}{189}\;h^{2}\eta_{m+6,n} \end{array}$$ (32)

$$\begin{array}{ccc} u_{m+5,n} & = & u_{m,n}+5h{u^{\prime }}_{m,n}+\frac{35225}{24192}\;h^{2}\eta_{m,n}+\frac{8375}{1344}\;h^{2}\eta_{m+1,n}+\frac{3125}{8064}\;h^{2}\eta_{m+2,n}+\frac{25625}{6048}\;h^{2}\eta_{m+3,n}\\ & & -\frac{625}{2688}\;h^{2}\eta_{m+4,n}+\frac{275}{576}\;h^{2}\eta_{m+5,n}-\frac{1375}{24192}\;h^{2}\eta_{m+6,n} \end{array}$$ (33)

$$\begin{array}{ccc} u_{m+6,n} & = & u_{m,n}+6h{u^{\prime }}_{m,n}+\frac{123}{70}\;h^{2}\eta_{m,n}+\frac{54}{7}\;h^{2}\eta_{m+1,n}+\frac{27}{35}\;h^{2}\eta_{m+2,n}+\frac{204}{35}\;h^{2}\eta_{m+3,n}\\ & & +\frac{27}{70}\;h^{2}\eta_{m+4,n}+\frac{54}{35}\;h^{2}\eta_{m+5,n} \end{array}$$ (34)

To determine the first derivatives of Implicit Six-point block solver, the values of *u*_*m*+1,*n*_, *u*_*m*+2,*n*_, *u*_*m*+3,*n*_, *u*_*m*+4,*n*_, *u*_*m*+5,*n*_, and *u*_*m*+6,*n*_ in (29)–(34) are substituted into (21)–(26) which produces;

$$\begin{array}{ccc} {u^{\prime }}_{m+1,n} & = & {u^{\prime }}_{m,n}+\frac{19087}{60480}\;h\eta_{m,n}+\frac{2713}{2520}\;h\eta_{m+1,n}-\frac{15487}{20160}\;h\eta_{m+2,n}+\frac{586}{945}\;h\eta_{m+3,n}-\frac{6737}{20160}\;h\eta_{m+4,n}\\ & & +\frac{263}{2520}\;h\eta_{m+5,n}-\frac{863}{60480}\;h\eta_{m+6,n} \end{array}$$ (35)

$$\begin{array}{ccc} u_{m+2,n}^{\prime } & = & {u^{\prime }}_{m,n}+\frac{1139}{3780}\;h\eta_{m,n}+\frac{94}{63}\;h\eta_{m+1,n}+\frac{11}{1260}\;h\eta_{m+2,n}+\frac{332}{945}\;h\eta_{m+3,n}-\frac{269}{1260}\;h\eta_{m+4,n}\\ & & +\frac{22}{315}\;h\eta_{m+5,n}-\frac{37}{3780}\;h\eta_{m+6,n} \end{array}$$ (36)

$$\begin{array}{ccc} {u^{\prime }}_{m+3,n} & = & {u^{\prime }}_{m,n}+\frac{137}{448}\;h\eta_{m,n}+\frac{81}{56}\;h\eta_{m+1,n}+\frac{1161}{2240}\;h\eta_{m+2,n}+\frac{34}{35}\;h\eta_{m+3,n}-\frac{729}{2240}\;h\eta_{m+4,n}\\ & & +\frac{27}{280}\;h\eta_{m+5,n}-\frac{29}{2240}\;h\eta_{m+6,n} \end{array}$$ (37)

$$\begin{array}{ccc} {u^{\prime }}_{m+4,n} & = & {u^{\prime }}_{m,n}+\frac{286}{945}\;h\eta_{m,n}+\frac{464}{315}\;h\eta_{m+1,n}+\frac{128}{315}\;h\eta_{m+2,n}+\frac{1504}{945}\;h\eta_{m+3,n}+\frac{58}{315}\;h\eta_{m+4,n}\\ & & +\frac{16}{315}\;h\eta_{m+5,n}-\frac{8}{945}\;h\eta_{m+6,n} \end{array}$$ (38)

$$\begin{array}{ccc} u_{m+5,n}^{\prime } & = & {u^{\prime }}_{m,n}+\frac{3715}{12096}\;h\eta_{m,n}+\frac{725}{504}\;h\eta_{m+1,n}+\frac{2125}{4032}\;h\eta_{m+2,n}+\frac{250}{189}\;h\eta_{m+3,n}+\frac{3875}{4032}\;h\eta_{m+4,n}\\ & & +\frac{235}{504}\;h\eta_{m+5,n}-\frac{275}{12096}\;h\eta_{m+6,n} \end{array}$$ (39)

$$\begin{array}{ccc} u_{m+6,n}^{\prime } & = & {u^{\prime }}_{m,n}+\frac{41}{140}\;h\eta_{m,n}+\frac{54}{35}\;h\eta_{m+1,n}+\frac{27}{140}\;h\eta_{m+2,n}+\frac{68}{35}\;h\eta_{m+3,n}+\frac{27}{140}\;h\eta_{m+4,n}\\ & & +\frac{54}{35}\;h\eta_{m+5,n}+\frac{41}{140}\;h\eta_{m+6,n} \end{array}$$ (40)

**Remark 1:** The Eqs (29)–(34) and (35)–(40) are the acquired Implicit Six-point Block Scheme (ISBS) required for approximating (1) directly without starting values or separate development of predictors.

**Remark 2:** There must be a non-singular square matrix Θ in Eq (9). If not, there wouldn’t be a solution.

**Remark 3:** As the analysis section demonstrates, the Eqs (29)–(34) and (35)–(40) that make up the ISBS have a consistent order of accuracy.

**Remark 4:** The continuous scheme (10) which has a coefficients in matrix form in (10) and (11) must be a continuous function, differentiable, and the limit must exist.

**Remark 5:** For a matrix Eq (9) to be valid, the number of parameters of the equations to be solved must coincide with the number of unknown.

## 3. The analysis of the ISBS

Next stage is to presents the preliminary of ISBS’s theoretical analysis, Convergence Analysis, Absolute Stability Region, and some useful definitions.

### 3.1 Preliminary of ISBS’s theoretical analysis

According to the established theorem by in Familua et al. and Jain et al. in references [43] and [57]), this subsection analyzed the order of accuracy, constants of errors, zero-stability, and finally the consistency of the ISBS. The linear operator listed below can be used to represent the Scheme (28) and its related variants.

$$\begin{array}{c} L\left[u\left(x\right);h\right]=U_{m}-\bar{F}\lambda_{0}+\bar{G}\lambda_{1}+h^{2}\left[\bar{H}\lambda_{2}+\bar{I}\lambda_{3}\right], \end{array}$$ (41)

$U_{m},\bar{F}\lambda_{0},\bar{G}\lambda_{1},\bar{H}\lambda_{2}$, and $\bar{I}\lambda_{3}$ have the above-mentioned typical meanings, and *u*(*m*) is continuously differentiable. Determining the similar terms in powers of *h* and *u*, and expanding *U*_*m*_, λ_2_, and λ_3_ in (41), correspondingly, in Taylor series concerning *x*_*n*_, provides,

$$\begin{array}{c} L\left[u\left(x\right);h\right]={\bar{C}}_{0}u(x)+{\bar{C}}_{1}hu^{\prime }(x)+{\bar{C}}_{2}h^{2}u^{\prime \prime }(x)+...+{\bar{C}}_{s}h^{s}u^{\left(s\right)}(x) \end{array}$$ (42)

where ${\bar{C}}_{s},s=1,2,...$

**Definition 3.1.1 (Order)** (Familua et *al.* [43])

The ISBS (29) and its linear operators are assigned an order *p* if ${\bar{C}}_{0}={\bar{C}}_{1}=...={\bar{C}}_{p}=0,{\bar{C}}_{p+2}\neq 0$.

**Definition 3.1.2 (Local Truncation Error)** (Jain et *al.* [58])

In Definition 3.1.1, the word ${\bar{C}}_{p+2}$ denotes the error constants that represent the local truncation error (18), as

$$\begin{array}{c} L.T.E.={\bar{C}}_{p+2}h^{p+2}u^{\left(p+2\right)}(x_{n})+Oh^{\left(p+3\right)} \end{array}$$ (43)

**Definition 3.1.3 (consistency)** (Ken **et al.** [59])

A consistency is defined as any LMM class where the order is larger than or equal to one.

**Definition 3.1.4 (zero-stability)** (Bruce [60])

A class of LMM is considered zero-stable if the roots of the differential equations under study do not exceed the order of the differential equations under consideration.

**Definition 3.1.5 (convergence)** (Lambert [61])

An LMM class is considered convergent if it is consistent and zero-stable.

**Definition 1.3.6 (Singular matrix)** (Lambert [61])

A singular matrix is a matrix with zero determinant.

**Definition 1.3.7 (Maximal order)** (Jain et *al.* [58])

The LM technique is said to be of maximum order if its order is 2*k* for even *k* and 2*k* − 1 for odd *k*.

**Definition 1.3.8 (Non-singular matrix)** (Jain et *al.* [58])

A non-singular matrix is one whose determinant does not equal zero.

**Definition 1.3.9 (P-stability)** (Ken **et al.** [59])

If an LMM’s periodicity interval is (0, ∞), it is considered P-stable.

**Definition 1.3.10 (Identity matrix)** (Jain et *al.* [58])

a matrix where all other entries are zero and the leading diagonal is one.

**Definition 1.3.11 (A-stability)** (Hericin [62])

If an LMM’s periodicity interval is (−∞, 0), it is considered A-stable.

**Definition 1.3.12 (Matrix)** (Hericin [62])

A rectangular array of integers that adheres to certain combination laws is called a matrix.

**Definition 1.3.13 (Inverse of Matrix)** (Ken **et al.** [59])

The inverse of a matrix A denoted by *A*^−1^ is given as $A^{-1}=\frac{adj(A)}{\left|A\right|}$. where adj(A) is the ad joint of matrix *A* and |*A*| is the determinant of the matrix.

**Definition 1.3.14 (Row matrix)** (John [63])

A matrix with a single row of elements.

**Definition 1.3.15 (Column matrix)** (John [63])

A matrix with a single column of elements.

**Definition 1.3.16 (Unit matrix)** (Hericin [62])

A matrix in which all the elements on a principal diagonal are equal and all the non-diagonal elements are equal to zero.

**Definition 1.3.17 (Null or Empty matrix)** (Bruce [60])

A matrix in which all its elements has zero.

#### 3.1.1 ISBS’s order and error parameters

The ISBS are analyzed using the procedure and approach described in **3.1.1**. Each of the (29)–(40) that made up the ISBS is looked at. Consequently, the ISBS has error constants ${\bar{C}}_{p+2}=C_{9}$ and is of order *p* = [7, 7, 7, 7, 7, 7, 7, 7, 7, 7]^*T*^ respectively.

$$\begin{array}{c} \begin{array}{c} {\bar{C}}_{9}={\left(6.65\times 10^{-3},1.64\times 10^{-2},2.57\times 10^{-2},3.50\times 10^{-2},4.48\times 10^{-2},5.14\times 10^{-2},\right)}^{T} \end{array} \end{array}$$

$$\begin{array}{c} \begin{array}{c} {\left(1.14\times 10^{-2},8.47\times 10^{-3},1.00\times 10^{-2},8.47\times 10^{-3},1.14\times 10^{-2},0\right)}^{T} \end{array} \end{array}$$

#### 3.1.2 Consistency of the ISBS

Applying definition of Consistency in (3.1.3), the ISBS (29)–(34) together with the derivative (35)–(40). should the method’s order be larger than or equal to one, it is considered consistent. Since the ISBS has order *p* = 7 > 1, it is consistent (Omole *et al.* *al.* [64]).

#### 3.1.3 ISBS’s zero stability

Similarly, the first characteristics polynomial of the ISBS, provided by (Fatunla [65]), may be used to establish the zero-stability of the ISBS.

$$\begin{array}{c} \Pi \left(q\right)=\text{det}(qU_{m}-\bar{F})=0 \end{array}$$ (44)

Thus,

$$\begin{array}{c} \Pi \left(z\right)=[q(\begin{array}{cccccc} 1 & 0 & 0 & 0 & 0 & 0\\ 0 & 1 & 0 & 0 & 0 & 0\\ 0 & 0 & 1 & 0 & 0 & 0\\ 0 & 0 & 0 & 1 & 0 & 0\\ 0 & 0 & 0 & 0 & 1 & 0\\ 0 & 0 & 0 & 0 & 0 & 1 \end{array})-(\begin{array}{cccccc} 0 & 0 & 0 & 0 & 0 & 1\\ 0 & 0 & 0 & 0 & 0 & 1\\ 0 & 0 & 0 & 0 & 0 & 1\\ 0 & 0 & 0 & 0 & 0 & 1\\ 0 & 0 & 0 & 0 & 0 & 1\\ 0 & 0 & 0 & 0 & 0 & 1 \end{array})]=0 \end{array}$$

$$\begin{array}{c} \Pi \left(q\right)=q^{5}\left(q-1\right)=0, \end{array}$$ (45)

solving for the values of *q* in (46) to obtain *q* = 0, 0, 0, 0, 0, 1. Hence, worth concluding that the ISBS is hence zero-stable.

#### 3.1.4 ISBS’s convergence analysis

As per the definition given in Definition *3.1.5*, the linear multistep method just has to exhibit consistency and zero-stability. Therefore, the ISBS’s zero-stability and consistency indicate that it is convergent throughout, so ending the proof; see Henrici [62].

#### 3.1.5 Absolute stability region of ISBS

Lastly, the method outlined in Lambert [61] and Yakubu *et al.* [66] is used to analyze and discuss the stability of the ISBS.

$$\begin{array}{c} M\left(z\right)=zB{\left(I-zA\right)}^{-1}U+V \end{array}$$ (46)

Not only the stability function but also;

$$\begin{array}{c} p(n,z)=det(-M(z)+nI) \end{array}$$ (47)

The formulae (29)–(34) for the stability characteristics were created as,

$$\begin{array}{c} \left[\begin{array}{c} Y\\ ---\\ Y_{i+1} \end{array}]=[\begin{array}{ccc} A & & U\\ --- & --- & ---\\ B & & V \end{array}][\begin{array}{c} h^{2}\eta \left(u\right)\\ ---\\ Y_{i-1} \end{array}\right] \end{array}$$ (48)

$$\begin{array}{c} Y_{i-1}=[\begin{array}{c} \;u_{m+1,n}\\ \;u_{m,n} \end{array}],Y_{i+1}=[\begin{array}{c} \;u_{m+1,n}\\ \;u_{m+6,n} \end{array}],V=[\begin{array}{cc} \;0 & 1\\ \;0 & 1 \end{array}],I=[\begin{array}{cc} \;1 & 0\\ \;0 & 1 \end{array}] \end{array}$$

**Fig 1:**
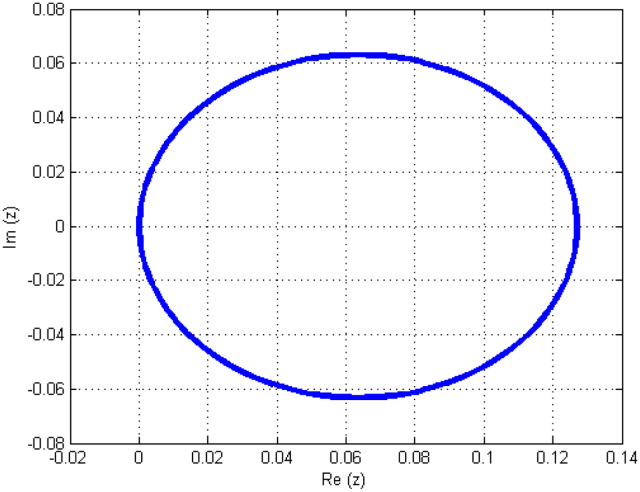
ISBS’s Stability domain.

Consequently, *n* denotes the roots of the ISBS’s first characteristics polynomial; a detailed presentation of the other parameters may be found in (Appendix B in S1 File). The parameters of A, B, U, V, M, and I (as they appear in Appendix C in S1 File) are then substituted into Eqs (47) and (48) to get the stability polynomial (49) and its first derivative (50). After that, the MATLAB (R2012a) environment is used to code this. In the text following, Fig 1 illustrates the stability of the ISBS.

$$\begin{array}{c} \eta (z)=(\varphi +\frac{2763}{280}\;z^{2}+\frac{507}{35}\;z-1)\varphi^{5} \end{array}$$ (49)

$$\begin{array}{c} \eta^{\prime }(z)=(\varphi +\frac{2763}{280}\;z^{2}+\frac{507}{35}\;z-1)\varphi^{5} \end{array}$$ (50)

The complex plane beyond the boxed image makes up the ISBS’s absolute stability area in Fig 1, which is P-stable since its interval falls within (0.13, 0), which is inside the defined interval for P-stability as contained and established in Definition 1.3.9 (∞, 0).

## 4. Computational procedure of PDEs originating from physcial system using ISBS

This section presents the process by which the ISBS is being applied to physical system problems. The variable *y* is discretized via a slightly similar approach of (Liu *et al.* [24] and Ramos & Vigorous [38]) as illustrated as follows;

$$\begin{array}{c} x_{j}=a+jh,\;\;j=0,1,\cdots ,M.,\;\Delta y=\frac{b-a}{M}\;y_{j}=c+jh,;\;j=0,1,\cdots ,N,h=\frac{d-c}{N}. \end{array}$$ (51)

*N* is the number of sub-intervals or iterations.

Thus, it follows that *j* = 0, ⋯, *M* for a fixed *x* in the interval [*a*, *b*], and *j* = 0, ⋯, *N* for a fixed *y* in the interval [*c*, *d*]. The difference operator approximates the spatial derivative and replaces it appropriately,

$$\begin{array}{c} \frac{\partial u}{\partial y}\approx \frac{u\left(x,y_{i+1}\right)-u\left(x,y_{i-1}\right)}{2\Delta y}, \end{array}$$ (52)

$$\begin{array}{c} \frac{\partial^{2}u}{\partial y^{2}}\approx [\frac{u\left(x,y_{i+1}\right)-2u\left(x,y_{i}\right)+u\left(x,y_{i-1},y\right)}{{\left(\Delta y\right)}^{2}}], \end{array}$$ (53)

The numerical approximation to *u*(*x*, *y*_*i*+1_) is denoted by *u*(*x*, *y*_*i*+1_).

Consequently, (1) has assumed the semi-discretized form displayed below.

$$\begin{array}{ccc} \frac{d^{2}u_{i,n}}{dx^{2}} & = & \frac{1}{a_{i,n}}[-b_{i,n}[\frac{u\left(x,y_{i+1}\right)-2u\left(x,y_{i}\right)+u\left(x,y_{i-1},y\right)}{{\left(\Delta y\right)}^{2}}]-p_{i,n}\frac{du_{i,n}}{dx}-\\ & & -r_{i,n}[\frac{u\left(x,y_{i+1}\right)-u\left(x,y_{i-1}\right)}{2\Delta y}]-k_{i,n}u_{i,n}+g_{i,n}], \end{array}$$ (54)

The non-homogeneous terms are represented by *g*, the step-size by *h*, and *A* represents the tridiagonal matrix generated by (54) (see to Appendix D in S1 File for details). Using Mathematical 11.0, which offers features such as Findroot for nonlinear problems and Nsolve for linear ones. Next, we applied the proposed ISBS to the resultant equations of ODEs with starting or boundary conditions Eq (54).

## 5. Numerical examples with ISBS application

This section presents the ISBS’s accuracy and convergence. We solved three numerical instances of partial differentials from the literature that originate from physical systems in two-dimensional space. In addition to comparing the mistakes created by various approaches that are currently in the literature, a comparison was done between the precise results and the numerical solution generated by ISBS. The findings are tabulated to highlight the accuracy of ISBS and its advantages over the already existing methods in the literatures. The proposed method, ISBS, computes its absolute error and compares its results with those of other methods that have been proposed previously, such as Yang *et al.* [67], Adeyefa and Omole [68], Lakestani & Dehghan [69], and Iqbal & Abass [70]. This discussion also includes the approaches’ outcomes. The Error obtained namely; Absolute error (AEs) is given by = Max | *a* − *b* |. It should be noted that *a* = *u*(*x*_*m*_, *y*_*m*_) is the exact solution whereas, *b* = *u*_*m*_(*y*_*n*_ is the approximate solution at the mesh point chosen.

## 5.1 Example 1

Consider the two-dimensional second-order elliptic differential equation which was examined by Yang *et al.* [67].

$$\begin{array}{c} \frac{\partial^{2}u}{\partial x^{2}}+\frac{\partial^{2}u}{\partial y^{2}}=\left(x^{2}+y^{2}\right)e^{xy},\;\text{for}\;0\leq x\leq 2,\;0\leq y\leq 1, \end{array}$$ (55)

in addition to the subsequent boundary requirements,

$$\begin{array}{ll} u(x,0)=1, & 0\leq x\leq 2\\ u(x,1)=e^{x}, & 0\leq x\leq 2\\ u(0,y)=1, & 0\leq y\leq 1\\ u(2,y)=e^{2y}, & 0\leq x\leq 1 \end{array}$$ (56)

The given problem (55) has an analytical solution as

$$\begin{array}{c} u\left(x,y\right)=e^{xy}. \end{array}$$ (57)

- ISBS—Implicit Six-point Block Scheme of uniform order 7 proposed in the present work.
- LIELM—Legendre Improved Extreme Learning Machine by Yang *et al.* [67]
- AEs—Absolute errors.

**Fig 2:**
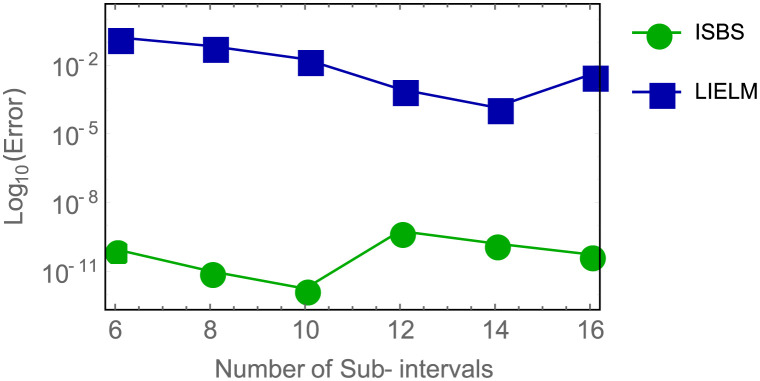
Logarithm curve of errors in ISBS versus LIELM for example 1.

In Table 1, columns 1, 2, and 3 illustrate, respectively, the outcomes of the theoretical solution or analytical solution, computed solution, and the absolute error for example 1 and the ISBS solution. It is evident that the computed solution’s findings converge to the theoretical solution. Similar to this, Table 2 compares the ISBS’s performance with LIELM to assess the ISBS’s accuracy in relation to LIELM, as reported by Yang *et al.* [67]. In Fig 2, we also show the logarithm plot comparing ISBS vs LIELM. As a result, ISBS data indicate higher productivity compared to LIELM.

**Table 1:** The numerical results for example 1.

N	Exact solution	ISBS solution	AE in ISBS
2	1.03174340749910280	1.03174340748469340	1.4409 × 10^−11^
4	1.01257845154063440	1.01257845155039820	9.7637 × 10^−12^
6	1.01387335406817300	1.01387335397299160	9.5181 × 10^−11^
8	1.00784309720644800	1.00784309719585850	1.0590 × 10^−11^
10	1.00501252085940100	1.00501252085757000	1.8310 × 10^−12^
12	1.00691278374453700	1.00691278433307920	5.8854 × 10^−10^
14	1.00511528351748440	1.00511528368699920	1.6951 × 10^−10^
16	1.00391388933834750	1.00391388939492900	5.6582 × 10^−11^
18	1.00314092239219570	1.00314092241527280	2.3077 × 10^−11^
20	1.00250312760579520	1.00250312761497740	9.1822 × 10^−12^

**Table 2:** Comparison of AEs in ISBS and LIELM for example 1.

N	Errors in ISBS	Error in LIELM
6	9.5181 × 10^−11^	1.6469 × 10^−01^
8	1.0590 × 10^−11^	6.7706 × 10^−02^
10	1.8310 × 10^−12^	1.8110 × 10^−02^
12	5.8854 × 10^−10^	8.8917 × 10^−04^
14	1.6951 × 10^−10^	1.4464 × 10^−04^
16	5.6582 × 10^−11^	3.7625 × 10^−03^

### 5.2 Example 2

Secondly, we take into consideration the two-dimensional second-order PDEs arising in Physical system studied by Yang *et al.* [67] and also Tsoulos *et al.* in reference [71].

$$\begin{array}{c} \frac{\partial^{2}u}{\partial x^{2}}+\frac{\partial^{2}u}{\partial y^{2}}=-2Sin\left(x\right)Cos\left(y\right),\;0\leq x\leq 1,\;0\leq y\leq 1, \end{array}$$ (58)

with boundary conditions

$$\begin{array}{c} u(x,0)=Sin(x),\;0\leq x\leq 1\\ u(x,1)=Sin(x)Cos(1),\;0\leq x\leq 1\\ u(0,y)=0,\;0\leq y\leq 1\\ u(1,y)=Sin(1)Cos(y),\;0\leq x\leq 1 \end{array}$$ (59)

The given exact solution is

$$\begin{array}{c} u(x,y)=Sin(x)Cos(y) \end{array}$$ (60)

- ISBS—Implicit Six-point Block Scheme of uniform order 7 proposed in the present work.
- LIELM—Legendre Improved Extreme Learning Machine by Yang *et al.* [67]
- CNN—Constructed Neutral Network produced by Tsoulos *et al.* [71]
- MAEs—Maximum Absolute Error

**Fig 3:**
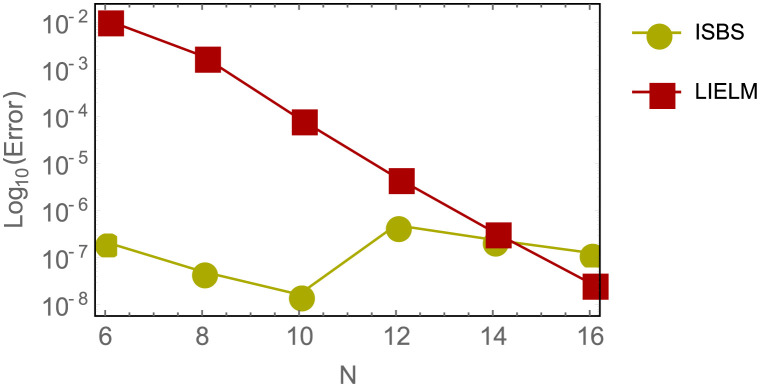
Logarithmic curve of errors in ISBS versus LIELM in for example 2, Shows the comparison analysis and performance of the ISBS and other existing methods in the cited literatures.

This section covers the ISBS numerical calculation results for example 2, which are presented in the Table 3 provided. Meanwhile, the assessment of AEs error in ISBS is made with LIELM proposed by Yang *et al.* in [67]. On the other hand, in Table 4, the MAEs of ISBS at *N* = 2 was determined and compared with other existing methods namely LIELM and CNN developed by Yang *et al.* [67] and Tsoulos *et al.* [71] respectively. It could be seen that in all the ISBS shows high-level of performance in terms of accuracy and convergence than other existing methods. The logarithm plot of Table 5 is also shown in Fig 3 to demonstrate the results graphically for easy analysis.

**Table 3:** Numerical result for example 2.

N	Exact solution	ISBS solution	AEs in ISBS
2	0.478489465369979100	0.478489049493883600	4.1588 × 10^−07^
4	0.247094768728200400	0.247094755255772100	1.3472 × 10^−08^
6	0.164669829139475100	0.164669615418555000	2.1372 × 10^−07^
8	0.124431307302325040	0.124431256843562420	5.0459 × 10^−08^
10	0.099708650872138790	0.099708634541371400	1.6331 × 10^−08^
12	0.082619333527632820	0.082619819444321240	4.8592 × 10^−07^
14	0.071187278914784780	0.071187517966933400	2.3905 × 10^−07^
16	0.062337366692613846	0.062337492660293650	1.2597 × 10^−07^
18	0.055882996075642600	0.055883070085200895	7.4010 × 10^−08^
20	0.049916708323414080	0.049916750915477440	4.2592 × 10^−08^

**Table 4:** Comparison of MAEs in ISBS with LIELM and CNN for example 2.

N	MAEs in ISBS	MAEs in LIELM	MAEs in CNN.
2	2.15 × 10^−11^	1.46 × 10^−06^	2.50 × 10^−05^

**Table 5:** Comparison of LIELM and ISBS absolute errors for example 2.

N	AEs in ISBS	AEsr in LIELM
6	2.1372 × 10^−07^	1.147 × 10^−02^
8	5.0459 × 10^−08^	1.9407 × 10^−03^
10	1.6331 × 10^−08^	9.0274 × 10^−05^
12	4.8592 × 10^−07^	5.0834 × 10^−06^
14	2.3905 × 10^−07^	3.5712 × 10^−07^
16	1.2597 × 10^−07^	2.8968 × 10^−08^

### 5.3 Example 3

Last but not least, Lakestani and Dehghan [69] solved the non-linear Klein-Gordon Equation with non-homogeneous term.

$$\begin{array}{c} \frac{\partial^{2}u}{\partial t^{2}}-\mu \frac{\partial^{2}u}{\partial x^{2}}+k\left(u\right)=g\left(x,t\right),\;\text{for}\;t>0\; \end{array}$$ (61)

It follows that; *g*(*x*, *t*) = 6*xt*(*x*^2^−*t*^2^) + *x*^6^*t*^6^, *μ* = 1, and *k*(*u*) = *u*^2^.

with either initial or any other similar boundary conditions on the interval [0, 1] below,

$$\begin{array}{c} u(x,0)=0,\\ u(x,1)=x^{3},\\ u(0,t)=0,\\ u(1,t)=t^{3},\\ u_{t}(x,0)=0,u_{x}(t,0)=0, \end{array}$$ (62)

The theoretical solution of (61) is given by,

$$\begin{array}{c} u\left(x,t\right)=t^{3}x^{3} \end{array}$$ (63)

- ISBS—Implicit Six-point Block Scheme of uniform order 7 proposed in the present work.
- FIBUM—Five-step Implicit Block Unification Method of order 6 constructed by Adeyefa & Omole [68].
- CWSCM—Chebyshev Wavelets Spectral Collocation Method developed by Iqbal & Abass [70].
- MFDCM—Modified Finite Difference Collocation Method examined by Lakestani & Dehghan [69].
- CM—Collocation method by Lakestani & Dehghan [69].

**Fig 4:**
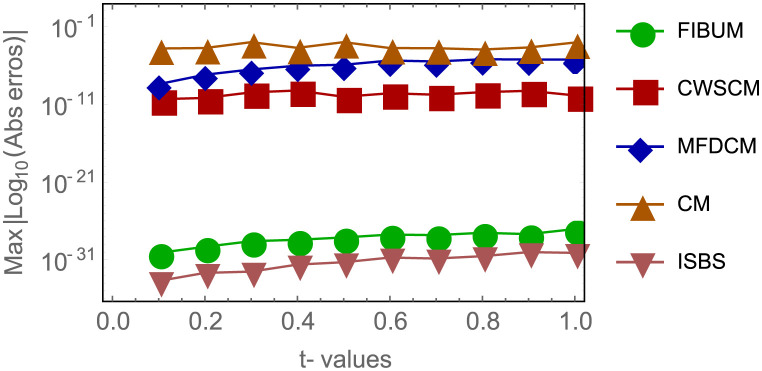
Comparison of AEs of different methods with ISBS for example 3, shows the comparison analysis and performance of the ISBS and other existing methods.

Next is to present the numerical results of example 3, which is a notable non-linear application problem arising from physical system. In Table 6, the computation of results of ISBS were presented namely, the analytical solution, the ISBS solution and the Absolute error. Likewise, Table 7 showcase the comparison of the absolute errors of the ISBS versus other similar existing recent methods in the literature. In particular, FIBUM of algebraic order six constructed by Adeyefa & Omole [68], CWSCM developed by Iqbal & Abass [70], MFDCM and CN both proposed by Lakestani & Dehghan [69]. It is obvious that ISBS of algebraic order seven outperformed FIBUM of algebraic order six, In the same vein, ISBS performed almost as thrice as much as the existing methods as shown in Table 7. The comparison of the absolute error is also shown by employing a logarithm plot for all the values of *t*, say *t* = 0.1, 0.2, …, 1.0. in Fig 4. It could be seen that Fig 4 shows the comparison analysis and performance of all the methods.

**Table 6:** The numerical results of ISBS for example 3.

t	Exact solution	ISBS solution	AEs in ISBS
0.1	9.702990000000002 × 10^−19^	9.702990000000003 × 10^−19^	1.93 × 10^−34^
0.2	7.762392000000001 × 10^−18^	7.762392000000002 × 10^−18^	2.11 × 10^−33^
0.3	2.619807300000000 × 10^−17^	2.619807300000000 × 10^−17^	3.08 × 10^−33^
0.4	6.209913600000004 × 10^−17^	6.209913600000002 × 10^−17^	2.47 × 10^−32^
0.5	1.212873749999999 × 10^−16^	1.212873750000000 × 10^−16^	4.93 × 10^−32^
0.6	2.095845840000002 × 10^−16^	2.095845840000000 × 10^−16^	1.97 × 10^−31^
0.7	3.328125569999999 × 10^−16^	3.328125570000000 × 10^−16^	1.48 × 10^−31^
0.8	4.967930880000004 × 10^−16^	4.967930880000001 × 10^−16^	2.96 × 10^−31^
0.9	7.073479710000002 × 10^−16^	7.073479710000003 × 10^−16^	9.86 × 10^−31^
1.0	9.702990000000009 × 10^−16^	9.702990000000000 × 10^−16^	7.89 × 10^−31^

**Table 7:** On the comparison of AEs of different methods with ISBS for example 3.

*t*	AEs in FIBUM	AEs in CWSCM	AEs in MFDCM	AEs in CM	AEs in ISBS
0.1	7.89 × 10^−31^	4.5 × 10^−11^	3.9 × 10^−09^	1.5 × 10^−04^	1.93 × 10^−34^
0.2	4.73 × 10^−30^	6.5 × 10^−11^	6.3 × 10^−08^	1.7 × 10^−04^	2.11 × 10^−33^
0.3	2.52 × 10^−29^	3.4 × 10^−10^	3.0 × 10^−07^	9.7 × 10^−04^	3.08 × 10^−33^
0.4	3.79 × 10^−29^	5.9 × 10^−10^	9.1 × 10^−07^	1.8 × 10^−04^	2.47 × 10^−32^
0.5	7.59 × 10^−29^	9.3 × 10^−11^	1.2 × 10^−06^	9.7 × 10^−04^	4.93 × 10^−32^
0.6	1.77 × 10^−28^	2.6 × 10^−10^	4.2 × 10^−06^	1.7 × 10^−04^	1.97 × 10^−31^
0.7	1.51 × 10^−28^	1.7 × 10^−10^	3.2 × 10^−06^	1.6 × 10^−04^	1.48 × 10^−31^
0.8	3.03 × 10^−28^	3.6 × 10^−10^	6.1 × 10^−06^	1.1 × 10^−04^	2.96 × 10^−31^
0.9	2.02 × 10^−28^	5.4 × 10^−10^	5.7 × 10^−06^	2.0 × 10^−04^	9.86 × 10^−31^
1.0	9.09 × 10^−28^	1.4 × 10^−10^	5.5 × 10^−06^	8.7 × 10^−04^	7.89 × 10^−31^

**Fig 5:**
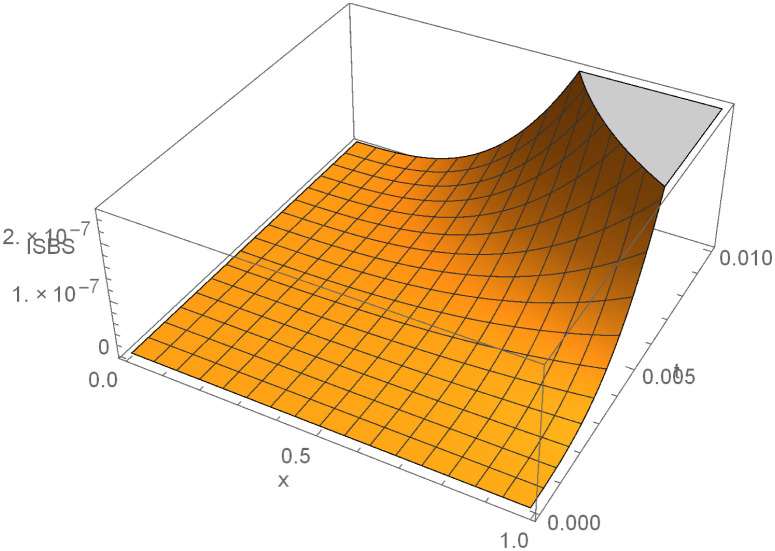
ISBI solution for example 3, Shows the ISBI computed solution in surface plot for example 3.

**Fig 6:**
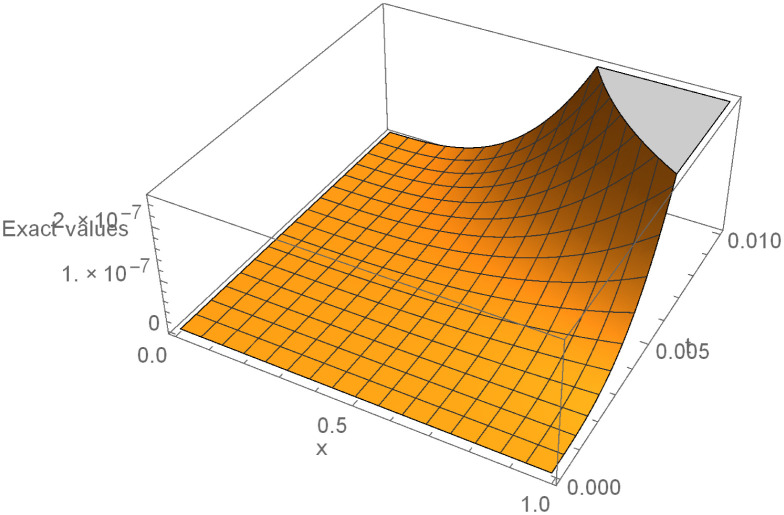
ISBI exact solution for example 3, Shows the ISBI exact solution in surface plot for example 3.

**Fig 7:**
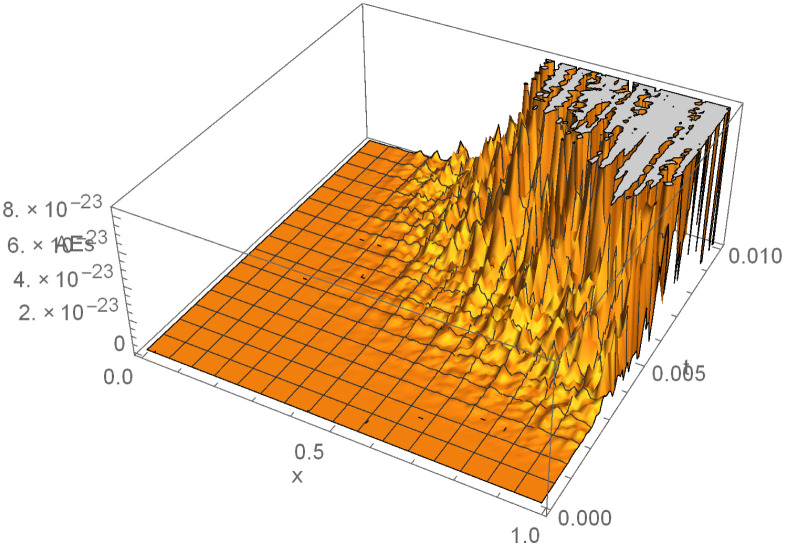
Absolute error (AEs) for example 3, Shows the Absolute error (AEs) in surface plot for example 3.

Finally, it is worth providing example 3 surface plot to show how the ISBS solution or computed solution, exact solution, and the absolute errors in Figs 5–7 behave in different ways. In general, it can be seen that the exact solution frequently converges to the ISBS solution. As a result, the absolute errors was relatively small. We come to the conclusion that the ISBI has a wider range of applications than the currently used methods described in the literature, is computationally dependable, and provides high-order precision.

## 6. Conclusion

In this report, the Power of implicit six-point block scheme had been Unveiled with the aim of advancing numerical approximation of two-dimensional PDEs in physical systems. To tackle many physical system partial differential equations (PDEs), we embarked on a comprehensive theoretical analysis and practical application of an innovative solution technique known as the Implicitly Stable Boundary Scheme (ISBS). Leveraging orthogonal polynomials as the trial functions and employing sophisticated collocation techniques, we meticulously crafted a robust framework for PDE resolution. One of the hallmark achievements of our study was the meticulous examination of the region of absolute stability using the boundary locus approach, revealing remarkable stability properties, as vividly illustrated in Fig 1. Notably, our investigation unveiled that the seventh-order convergence ISBS is not only zero-stable but exhibits exceptional consistency, laying a solid foundation for its widespread applicability. Focusing on semi-discretizing the governing equations by substituting the y-function or time-dependent derivatives, we transformed the complex PDEs into ordinary differential equations (ODEs), ripe for ISBS resolution. Through rigorous testing across three significant physics and engineering applications, our study unveiled compelling insights meticulously tabulated for clarity. In our pursuit of numerical precision, we meticulously compared the ISBS solutions with theoretical counterparts, presenting comprehensive numerical results in Tables 1, 3 and 6. Furthermore, we conducted a thorough evaluation against other established techniques, showcased in Tables 2, 4, 5 and 7, with logarithmic plots in Figs 2–4 providing visual clarity. The culmination of our efforts is exemplified in Figs 5–7, where surface plots for case 3 vividly depict the robustness and accuracy of the ISBS approach. The profound alignment between our findings and alternative methodologies underscores the efficacy of employing orthogonal polynomials as approximate functions in addressing one and two-dimensional physical system problems. Looking ahead, our future endeavours will be directed towards advancing the numerical solution of higher-order PDEs, accommodating varying conditions and variable step-sizes, further solidifying the practical utility and versatility of ISBS in tackling complex physical system dynamics.

## Supporting information

S1 File (PDF)

## References

[1] Ahsan M., Siraj-ul-Islam, & Hussain I. (2018). Haar wavelets multi-resolution collocation analysis of unsteady inverse heat problems. *Inverse Problems in Science and Engineering*, 27(11), 1498–1520. doi: 10.1080/17415977.2018.1481405

[2] Wang T., and Guo B. (2011). Unconditional convergence of two conservative compact difference schemes for non-linear Schrodinger equation in one dimension. *Sci. Sin. Math.* 2011, 41, 207–233. doi: 10.1360/012010-846

[3] Arif M. S., Shatanawi W., & Nawaz Y. (2023). Modified Finite Element Study for Heat and Mass Transfer of Electrical MHD Non-Newtonian Boundary Layer Nanofluid Flow. *Mathematics*, 11(4), 1064. doi: 10.3390/math11041064

[4] Arif M. S., Abodayeh K., & Nawaz Y. (2022). The modified finite element method for heat and mass transfer of unsteady reacting flow with mixed convection. *Frontiers in Physics*, 10, 952787. doi: 10.3389/fphy.2022.952787

[5] Adoghe L. O., Omole E. O., & Fadugba S. E. (2022). Third derivative method for solving stiff system of ordinary differential equations. *International Journal of Mathematics in Operational Research*, 23(3), 412. doi: 10.1504/IJMOR.2022.127382

[6] Nawaz Y., Arif M. S., Abodayeh K., & Bibi M. (2022). Finite Element Method for Non-Newtonian Radiative Maxwell Nanofluid Flow under the Influence of Heat and Mass Transfer. *Energies*, 15(13), 4713. doi: 10.3390/en15134713

[7] Animasaun, I. L., Shah, N. A., Wakif, A., Mahanthesh, B., Sivaraj, R., & Koriko, O. K. (2022). *Ratio of Momentum Diffusivity to Thermal Diffusivity: Introduction*, *Meta-analysis*, *and Scrutinization*. Chapman and Hall/CRC. New York. ISBN-13: 978-1032108520, ISBN-10: 1032108525, ISBN9781003217374. 10.1201/9781003217374

[8] Devnath S., Khan K., & Akbar M. A. (2023). Numerous analytical wave solutions to the time-fractional unstable nonlinear Schrodinger equation with beta derivative. *Partial Differential Equations in Applied Mathematics*, 8, 100537. doi: 10.1016/j.padiff.2023.100537

[9] Ahsan M., Ahmad I., Ahmad M., & Hussian I. (2019). A numerical Haar wavelet-finite difference hybrid method for linear and non-linear Schrodinger equation. *Mathematics and Computers in Simulation*, 165, 13–25. doi: 10.1016/j.matcom.2019.02.011

[10] Wang F., Animasaun I. L., Al-Mdallal Q. M., Saranya S., & Muhammad T. (2023). Dynamics through three-inlets of t-shaped ducts: Significance of inlet velocity on transient air and water experiencing cold fronts subject to turbulence. *International Communications in Heat and Mass Transfer*, 148, 107034. doi: 10.1016/j.icheatmasstransfer.2023.107034

[11] Rufai M. A., Shokri A., & Omole E. O. (2023). A One-Point Third-Derivative Hybrid Multistep Technique for Solving Second-Order Oscillatory and Periodic Problems. *Journal of Mathematics*, 2023, 1–12. doi: 10.1155/2023/2343215

[12] Nawaz Y., & Arif M. S. (2020). Modified class of explicit and enhanced stability region schemes: Application to mixed convection flow in a square cavity with a convective wall. *International Journal for Numerical Methods in Fluids*, 93(6), 1759–1787. doi: 10.1002/fld.4951

[13] Ramakrishna O., Falodun B. O., Akinremi O. J., Omole E. O., Ismail A. S., & Amoyedo F. E. (2024). Thermodynamics of variable thermophysical properties of non-Newtonian fluids with the exploration of antiviral and antibacterial mechanisms using silver nanoparticles. *International Journal of Thermofluids*, 22, 100648. doi: 10.1016/j.ijft.2024.100648

[14] El Misilmani H. M., Kabalan K. Y., Abou-Shahine M. Y. and Al-Husseini M. (2015). A Method of Moment Approach in Solving Boundary Value Problems, *Journal of Electromagnetic Analysis and Applications*, 7, 61–65. 10.4236/jemaa.2015.73007

[15] Quarteroni, A., and Valli, A. (1994). Numerical Approximation of Partial Differential Equations. In Springer Series in Computational Mathematics. Springer Berlin Heidelberg. https://doi.org/doi:10.1007/978-3-540-85268-1

[16] Le Dret, H., & Lucquin, B. (2016). Partial Differential Equations: Modeling, Analysis and Numerical Approximation. In International Series of Numerical Mathematics. Springer International Publishing. https://doi.org/doi:10.1007/978-3-319-27067-8

[17] Bergounioux, M., Haberkorn, T., & Privat, Y. (2016). An optimal control approach to photoacoustic tomography. 2016 IEEE 55th Conference on Decision and Control (CDC). https://doi.org/doi:10.1109/cdc.2016.7798497

[18] Gunzburger, M. D. (2003). Perspectives in flow control and optimization, SIAM. https://doi.org/doi:10.1137/1.9780898718720

[19] Bredies, K., Clason, C., Kunisch, K., and von Winckel, G., (2013). Control and Optimization with PDE Constraints, Birkhuauser Verlag, Basel Editors.

[20] Neittaanmaki, P. and Tiba, D. (1994). Optimal Control of Nonlinear Parabolic Systems: Theory, Algorithms, and Applications, Taylor & Francis.

[21] Leugering, G., Benner, P., Engell, S., Griewank, A., Harbrecht, H., Hinze, M., et al. editors (2014). Trends in PDE Constrained Optimization, Springer International Publishing, Switzerland.

[22] Aubert G. and Kornprobst P. (2006). Mathematical problems in image processing, Springer, New York.

[23] Debnath L. (2012). Nonlinear partial differential equations for scientists and engineers. Springer, New York.

[24] Liu X., Ahsan M., Ahmad M., Nisar M., Liu X., Ahmad I., et al. (2021). Applications of Haar Wavelet-Finite Difference Hybrid Method and Its Convergence for Hyperbolic Nonlinear Schrodinger Equation with Energy and Mass Conversion. *Energies*, 14, 7831. doi: 10.3390/en14237831

[25] Raslan K. R., Ali K. K., and Al-Bayatti H.M. Y (2021). Construct Extended Cubic B-Splines in n-Dimensional for Solving n-Dimensional Partial Differential Equations. *Applied Mathematics & Information Sciences*, 15(5), 599–611. doi: 10.18576/amis/150508

[26] Mirzaee F., Rezaei S., & Samadyar N. (2021). Numerical solution of two-dimensional stochastic time-fractional Sine-Gordon equation on non-rectangular domains using finite difference and meshfree methods. *Engineering Analysis with Boundary Elements*, 127, 53–63. doi: 10.1016/j.enganabound.2021.03.009

[27] Lakestani M. and Dehghan M. (2010). Collocation and Finite Difference-Collocation Methods for the Solution of Nonlinear Klein-Gordon Equation. *Computer Physics Communications*, 181, 1392–1401. doi: 10.1016/j.cpc.2010.04.006

[28] Shiralashetti S. C., Kantli M. H., and Deshi A. B. (2018). Biorthogonal wavelet-based full-approximation schemes for the numerical solution of elasto-hydrodynamic lubrication problems. *Journal of Mathematical Modeling*, 6(1), 105–122.

[29] Juraev D. A. and Gasimov Y. S. (2022). On the regularization Cauchy problem for matrix factorizations of the Helmholtz equation in a multidimensional bounded domain. *Azerbaijan Journal of Mathematics*, 12(1), 142–161.

[30] Butcher J. C., & Wanner G. (1996). Runge-Kutta methods: some historical notes. *Applied Numerical Mathematics*, 22(1-3), 113–151. doi: 10.1016/S0168-9274(96)00048-7

[31] Gragg W. B., & Stetter H. J. (1964). Generalized multistep predictor-corrector methods. *Journal of the ACM (JACM)*, 11(2), 188–209. doi: 10.1145/321217.321223

[32] Garrido I., Lee B., Fladmark G., & Espedal M. (2006). Convergent iterative schemes for time parallelization. *Mathematics of Computation*, 75(255), 1403–1428. doi: 10.1090/S0025-5718-06-01832-1

[33] Diamantakis M., Wood N., & Davies T. (2006). An improved implicit predictor-corrector scheme for boundary layer vertical diffusion. *Quarterly Journal of the Royal Meteorological Society: A journal of the Atmospheric Sciences*, *Applied Meteorology and Physical Oceanography*, 132(616), 959–978. doi: 10.1256/qj.05.37

[34] Su X., and Zhou Y. (2022). A Fast High-Order Predictor–Corrector Method on Graded Meshes for Solving Fractional Differential Equations. *Fractal Fract.*, 6, 516. doi: 10.3390/fractalfract6090516

[35] Shokri A., and Saadat H. (2016). P-stability, TF and VSDPL technique in Obrechkoff methods for the numerical solution of the Schrodinger equation, Bull. *Iranian Math. Soc.*, 42(3), pp. 687–706.

[36] Awoyemi D. O. (2003). A p-stable linear multistep method for solving third order ordinary differential equations. *Int.*, *J. Compt*, *math.*, 80(1): 85–91.

[37] Sunday S. J., Shokri A., and Marian D. (2022). Variable Step Hybrid Block Method for the Approximation of Kepler Problem. *Fractal Fract.*, 6, 343. doi: 10.3390/fractalfract6060343

[38] Ramos H., & Vigo-Aguiar J. (2007). An almost L-stable BDF-type method for the numerical solution of stiff ODEs arising from the method of lines. *Numerical Methods for Partial Differential Equations*, 23(5), 1110–1121. doi: 10.1002/num.20212

[39] Ngwane F. F., & Jator S. N. (2017). A Trigonometrically Fitted Block Method for Solving Oscillatory Second-Order Initial Value Problems and Hamiltonian Systems. *International Journal of Differential Equations*, 2017, 1–14. doi: 10.1155/2017/9293530

[40] Modebei M. I., Adeniyi R. B, and Jator S. N. (2020). Numerical approximation of fourth-order PDEs using block unification method. *Journal of the Nigerian Mathematical society*, 39(1), 47–68.

[41] Jator S. N. (2015). Block Unification Scheme for Elliptic, Telegraph, and Sine-Gordon Partial Differential Equations. *American Journal of Computational Mathematics*, 5, 175–185. doi: 10.4236/ajcm.2015.52014

[42] Olaiya O. O., Oduwole H. K., and Odeyemi J. K. (2019). Numerical solution of Black-Scholes Partial Differential Equation Using Direct Solution of Second-Order Ordinary Differential Equation With Two–Step Hybrid Block Method Of Order Seven, 14(2) 23–29.

[43] Familua A. B., Omole E. O., and Ukpebor L. A. (2022). A Higher-order Block Method for Numerical Approximation of Third-order Boundary Value Problems in ODEs. *Journal of the Nigerian Society of Physical Sciences*, 4(3), 706. doi: 10.46481/jnsps.2022.706

[44] Farkas M., & Deconinck B. (2023). Solving the heat equation with variable thermal conductivity. *Applied Mathematics Letters*, 135, 108395. doi: 10.1016/j.aml.2022.108395

[45] Animasaun, I. L., Shah, N. A., Wakif, A., Mahanthesh, B., Sivaraj, R., & Koriko, O. K. (2022). *Ratio of Momentum Diffusivity to Thermal Diffusivity: Introduction*, *Meta-analysis*, *and Scrutinization*. Chapman and Hall/CRC. New York. ISBN-13: 978-1032108520, ISBN-10: 1032108525, ISBN9781003217374. 10.1201/9781003217374.

[46] Ullah N., Asjad M. I., Hussanan A., Akgul A., Alharbi W. R., Algarni H., & Yahia I. S. (2023). Novel waves structures for two nonlinear partial differential equations arising in the nonlinear optics via Sardar-subequation method. *Alexandria Engineering Journal*, 71, 105–113. doi: 10.1016/j.aej.2023.03.023

[47] Salahudin N. A., Roslee N., Pakkal N. Z., Zokree S., & Saipan H. F. (2023). Simulation and visualization of wave equation. *Data Analytics and Applied Mathematics (DAAM)*, 49–54.

[48] Rehman S. U., Ahmad J., & Muhammad T. (2023). Dynamics of novel exact soliton solutions to Stochastic Chiral Nonlinear Schrodinger Equation. *Alexandria Engineering Journal*, 79, 568–580. doi: 10.1016/j.aej.2023.08.014

[49] Khan A., Saifullah S., Ahmad S., Khan M. A., & Rahman M. U. (2023). Dynamical properties and new optical soliton solutions of a generalized nonlinear Schrodinger equation. *The European Physical Journal Plus*, 138(11), 1059. doi: 10.1140/epjp/s13360-023-04697-5

[50] Johnson, P. J. (Ed.). (2021). Navier-Stokes Equations and their Applications. Nova Science Publishers. 10.52305/ujuz9424.

[51] Fisher M., Nocedal J., Tremolet Y., & Wright S. J. (2009). Data assimilation in weather forecasting: a case study in PDE-constrained optimization. *Optimization and Engineering*, 10(3), 409–426. doi: 10.1007/s11081-008-9051-5

[52] Jameson A., Martinelli L., & Pierce N. A. (1998). Optimum aerodynamic design using the Navier-Stokes equations. *Theoretical and computational fluid dynamics*, 10(1), 213–237. doi: 10.1007/s001620050060

[53] Thakare, N. K., Karande, B. K., & Karande, K. (1973). Some properties of orthogonal polynomials related to Hermite polynomials. Bulletin mathematique de la Societe des Sciences Mathematiques de la Republique Socialiste de Roumanie, 57–69.

[54] Adeyefa E. O., Omole E. O., & Shokri A. (2023). Numerical solution of second-order nonlinear partial differential equations originating from physical phenomena using Hermite based block methods. *Results in Physics*, 46, 106270. doi: 10.1016/j.rinp.2023.106270

[55] Dattoli G., & Licciardi S. (2023). Monomiality and a New Family of Hermite Polynomials. *Symmetry*, 15(6), 1254. doi: 10.3390/sym15061254

[56] Salzer H. E., Zucker R., and Capuano R. (1952). Table of the zeros and weight factors of the first twenty Hermite polynomials, *J. Research Nat. Bur. Standards* 48, 111–116. doi: 10.6028/jres.048.016

[57] Milne W. E. (1953). *Numerical solution of differential equations*, New York: John Wiley and Sons.

[58] Jain, M. K., Iyengar, S. K., and Jain, R. K. (2007). *Numerical Methods for Scientific and Engineering Computation*, fifth ed., pp. 282–283.

[59] Ken, Y. L., Ismail, I. F., and Suleiman, M. (2011). Block Methods for Special Second Order ODEs, PhD Thesis, Universiti Putra Malaysia.

[60] Bruce, E. S. (2007). The Computable Differential Equation, California State University Northridge, pp. 120–121.

[61] Lambert, J. D. (1973). *Computational Methods in Ordinary Differential Equations*, John Wiley & Sons Inc.

[62] Henrici, P. (1972). *Discrete variable method in ordinary differential equations*, John Wiley & Sons New York

[63] John, R. D. (1996). Numerical Methods for Differential Equations, New York, pp. 1-2.

[64] Omole, E.O., Adeyefa, E.O., Ayodele, V.I., Shokri, A., and Wang, Y. (2023). *Ninth-order Multistep Collocation Formulas for Solving Models of PDEs Arising in Fluid Dynamics: Design and Implementation Strategies*, Axioms 12, 891. 10.3390/axioms12090891.

[65] Fatunla, S. O. (1988). *Numerical methods for initial value problems in ordinary differential equations*, Academic press inc. Harcourt Brace Jovanovich Publishers, New York.

[66] Yakubu D. G., Aminu M., Tumba P. and Abdulhameed M. (2018). An efficient family of second derivative Runge-Kutta collocation methods for oscillatory systems, *Journal of the Nigerian Mathematical Society*, 37(2), 111–138.

[67] Yang Y., Hou, M., Sun, H., Zhang T., Weng, F., and Luo, J. (2019). Neural network algorithm based on Legendre improved extreme learning machine for solving elliptic partial differential equations. Soft Computing Methodologies and Application.

[68] Adeyefa E. O. and Omole E. O. (2023). A Five-step Continuous Implicit Block Unification Block Method for Second-order Elliptic Partial Differential Equations. International Journal of Mathematics in Operation Research, 24(3), 360–386. doi: 10.1504/IJMOR.2023.129482

[69] Lakestani M. and Dehghan M. (2010). Collocation and Finite Difference-Collocation Methods for the Solution of Nonlinear Klein-Gordon Equation. Computer Physics Communications, 181, 1392–1401. doi: 10.1016/j.cpc.2010.04.006

[70] Iqbal J., and Abass R. (2016). Numerical Solution of Klein/Sine-Gordon Equations by Spectral Method Coupled with Chebyshev Wavelets. Applied Mathematics, 7, 2097–2109. 10.4236/am.2016.717167

[71] Tsoulos I. G., Gavrilis D., and Glavas E. (2009). Solving differential equations with constructed neural networks. *Neuro Computing*, 72, pp. 2385–2391.

