# Supplementary material for: Unveiling the Power of Implicit Six-Point Block Scheme: Advancing numerical approximation of two-dimensional PDEs in physical systems
Source: PLoS One. 2024 May 16;19(5):e0301505. doi: 10.1371/journal.pone.0301505 (PMC11098417; doi:10.1371/journal.pone.0301505)
Supplement: S1 File — (PDF) [file pone.0301505.s001.pdf]

**Appendix A**

$$\zeta_0 = u_{m,n}, \zeta_1 = -\frac{1}{120960h}(28549\,h^2\eta_{m,n} + 57750\,h^2\eta_{m+1,n} - 51453\,h^2\eta_{m+2,n} + 42484\,h^2\eta_{m+3,n} - 23109\,h^2\eta_{m \ldots}$$ [illegible]

$$+ 7254\,h^2\eta_{m+5,n} - 995\,h^2\eta_{m+6,n} + 120960\,u_{m,n} - 120960\,u_{m+1,n}), \zeta_2 = \frac{1}{2}\eta_{m,n},$$

$$\zeta_3 = -\frac{1}{360h}(147\,\eta_{m,n} - 360\,\eta_{m+1,n} + 450\,\eta_{m+2,n} - 400\,\eta_{m+3,n} + 225\,\eta_{m+4,n} - 72\,\eta_{m+5,n} + 10\,\eta_{m+6,n}),$$

$$\zeta_4 = \frac{1}{4320h^2}(812\,\eta_{m,n} - 3132\,\eta_{m+1,n} + 5265\,\eta_{m+2,n} - 5080\,\eta_{m+3,n} + 2970\,\eta_{m+4,n} - 972\,\eta_{m+5,n} + 137\,\eta_{m+6,n}),$$

$$\zeta_5 = -\frac{1}{960h^3}(49\,\eta_{m,n} - 232\,\eta_{m+1,n} + 461\,\eta_{m+2,n} - 496\,\eta_{m+3,n} + 307\,\eta_{m+4,n} - 104\,\eta_{m+5,n} + 15\,\eta_{m+6,n}),$$

$$\zeta_6 = \frac{1}{4320h^4}(35\,\eta_{m,n} - 186\,\eta_{m+1,n} + 411\,\eta_{m+2,n} - 484\,\eta_{m+3,n} + 321\,\eta_{m+4,n} - 114\,\eta_{m+5,n} + 17\,\eta_{m+6,n}),$$

$$\zeta_7 = -\frac{1}{10080h^5}(7\,\eta_{m,n} - 40\,\eta_{m+1,n} + 95\,\eta_{m+2,n} - 120\,\eta_{m+3,n} + 85\,\eta_{m+4,n} - 32\,\eta_{m+5,n} + 5\,\eta_{m+6,n}),$$

$$\zeta_8 = \frac{1}{40320h^6}(\eta_{m,n} - 6\,\eta_{m+1,n} + 15\,\eta_{m+2,n} - 20\,\eta_{m+3,n} + 15\,\eta_{m+4,n} - 6\,\eta_{m+5,n} + \eta_{m+6,n})$$

**Appendix B**

$$B = \begin{bmatrix} \frac{28549}{120960} & \frac{275}{576} & -\frac{5717}{13440} & \frac{10621}{30240} & -\frac{7703}{40320} & \frac{403}{6720} & -\frac{199}{24192} \\ \frac{123}{70} & \frac{54}{7} & \frac{27}{35} & \frac{204}{35} & \frac{27}{70} & \frac{54}{35} & 0 \end{bmatrix}$$

$$A = \begin{bmatrix} 0 & 0 & 0 & 0 & 0 & 0 & 0 \\ \frac{28549}{120960} & \frac{275}{576} & -\frac{5717}{13440} & \frac{10621}{30240} & -\frac{7703}{40320} & \frac{403}{6720} & -\frac{199}{24192} \\ \frac{1027}{1890} & \frac{194}{105} & -\frac{8}{9} & \frac{788}{945} & -\frac{97}{210} & \frac{46}{315} & -\frac{19}{945} \\ \frac{759}{896} & \frac{1485}{448} & -\frac{2403}{4480} & \frac{45}{32} & -\frac{3267}{4480} & \frac{513}{2240} & -\frac{141}{4480} \\ \frac{1088}{945} & \frac{1504}{315} & -\frac{8}{105} & \frac{2624}{945} & -\frac{8}{9} & \frac{32}{105} & -\frac{8}{189} \\ \frac{35225}{24192} & \frac{8375}{1344} & \frac{3125}{8064} & \frac{25625}{6048} & -\frac{625}{2688} & \frac{275}{576} & -\frac{1375}{24192} \\ \frac{123}{70} & \frac{54}{7} & \frac{27}{35} & \frac{204}{35} & \frac{27}{70} & \frac{54}{35} & 0 \end{bmatrix}, M = \begin{bmatrix} 1 & 0 & 0 & 0 & 0 & 0 \\ 0 & 1 & 0 & 0 & 0 & 0 \\ 0 & 0 & 1 & 0 & 0 & 0 \\ 0 & 0 & 0 & 1 & 0 & 0 \\ 0 & 0 & 0 & 0 & 1 & 0 \\ 0 & 0 & 0 & 0 & 0 & 1 \end{bmatrix}$$

$$U = \begin{bmatrix} 0 & 1 \\ 0 & 1 \\ 0 & 1 \\ 0 & 1 \\ 0 & 1 \\ 0 & 1 \end{bmatrix}, Y = \begin{bmatrix} u_{m+1,n} \\ u_{m+2,n} \\ u_{m+3,n} \\ u_{m+4,n} \\ u_{m+5,n} \\ u_{m+6,n} \end{bmatrix}, \eta(y) = \begin{bmatrix} \eta_{m+1,n} \\ \eta_{m+2,n} \\ \eta_{m+3,n} \\ \eta_{m+4,n} \\ \eta_{m+5,n} \\ \eta_{m+6,n} \end{bmatrix}$$

**Appendix C**

$$T = \begin{pmatrix} \frac{-2}{(\Delta y)^2} & \frac{1}{(\Delta y)^2} & 0 & \cdots & \cdots & 0 & 0 & 0 \\ \frac{1}{(\Delta y)^2} & \frac{-2}{(\Delta y)^2} & \frac{1}{(\Delta y)^2} & \cdots & \cdots & 0 & 0 & 0 \\ 0 & \frac{1}{(\Delta y)^2} & \frac{-2}{(\Delta y)^2} & \cdots & \cdots & 0 & 0 & 0 \\ \vdots & \vdots & \vdots & \vdots & \cdots & \vdots & \cdots & \cdots \\ \\ \vdots & \vdots & \vdots & \vdots & \cdots & \vdots & \cdots & \cdots \\ 0 & 0 & 0 & \cdots & \cdots & \frac{-2}{(\Delta y)^2} & \frac{1}{(\Delta y)^2} & 0 \\ 0 & 0 & 0 & \cdots & \vdots & \frac{1}{(\Delta y)^2} & \frac{-2}{(\Delta y)^2} & \frac{1}{(\Delta y)^2} \\ 0 & 0 & 0 & \vdots & \cdots & 0 & \frac{1}{(\Delta y)^2} & \frac{-2}{(\Delta y)^2} \end{pmatrix}$$
